# Three-dimensional surface printing method for interconnecting electrodes on opposite sides of substrates

**DOI:** 10.1038/s41598-020-75556-x

**Published:** 2020-10-29

**Authors:** Md. Khalilur Rahman, Seong-jun Kim, Thanh Huy Phung, Jin-Sol Lee, Jaeryul Yu, Kye-Si Kwon

**Affiliations:** 1 Department of Electronic Materials and Devices Engineering, Soonchunhyang University, 22, Soonchunhyang-ro, Asan City, Chungnam 31538 South Korea; 2 Department of Physics, Comilla University, Cumilla, 3506 Bangladesh; 3 Global Technology Center, Samsung Electronics Co. Ltd, Suwon, South Korea; 4 Department of Mechanical Engineering, Soonchunhyang University, 22, Soonchunhyang-ro, , Asan City, Chungnam 31538 South Korea

**Keywords:** Engineering, Mechanical engineering

## Abstract

As the application of the direct printing method becomes diversified, printing on substrates with non-flat surfaces is increasingly required. However, printing on three-dimensional surfaces suffers from a number of difficulties, which include ink flow due to gravity, and the connection of print lines over sharp edges. This study presents an effective way to print a fine pattern (~ 30 μm) on three different faces with sharp edge boundaries. The method uses a deflectable and stretchable jet stream of conductive ink, which is produced by near-field electrospinning (NFES) technique. Due to added polymer in the ink, the jet stream from the nozzle is less likely to be disconnected, even when it is deposited over sharp edges of objects. As a practical industrial application, we demonstrate that the method can be effectively used for recent display applications, which require the connection of electrical signal and power on both sides of the glass. When the total length of printed lines along the ‘Π’ shaped glass surfaces was 1.2 mm, we could achieve the average resistance of 0.84 Ω.

## Introduction

Direct printing methods have been widely used as an additive manufacturing tool. By using printing methods, functional materials can be directly deposited on the target location, and thereby the manufacturing cost can be significantly reduced^1^. So far, most direct printing methods have been implemented on substrates with flat surface. On the other hand, recent industry application demands the printing capabilities on substrates with non-flat surfaces^1,2^. For implementation, the variation of stand-off distance should be minimized during the printing process for uniform and accurate droplet placement on non-flat substrates. As a result, printing on the non-flat surface requires three-dimensional (3D) motion control, while most 3D surface printing applications have been limited to surfaces of low curvature^3^. In the case of using conventional methods for printing on surfaces of high curvature, the control of 3D motion could be difficult, and the process can be very slow, with poor deposition quality.

Recently, glass has been widely used as a transparent substrate for display devices, such as touch screen panels, flat-panel TVs, and computer monitors. Here, electrical circuits and functional materials have been patterned on both sides of the glass. These double-layered patterns need connections for the proper operation of the device^4^. However, unlike printed circuit board (PCB) substrates, via holes for the connection of double-sided circuits are difficult to make, due to the brittleness of glass. Laser drilling could be used for making via holes. However, the process consumes a huge amount of energy and time^5,6^.

In display application, most electrode pads for connection purpose are located in the bezel, the boundary area of the glass substrate. The recent trend for monitor screens demands larger and wider visible area, which leads to a narrower bezel. As a result, the connection of circuits has become more difficult. So far, most connecting lines have been fabricated by using a photolithography process on each side. However, this is a costly process, and if the line connection over the sharp edges is required, the production yield could be very low, with lot of defects near the sharp glass edges. In order to solve these problems, there has been strong demand for direct printing methods to replace existing methods. However, most of the direct printing methods have been limited to surfaces with relatively large radius of curvature^2,7^, for the following reasons: (1) Printed ink could flow down vertical faces due to the effect of gravity, unless the ink viscosity is very high. (2) The printing speed could be very slow, in order for the nozzle to follow such irregular surfaces with extremely large curvature. (3) In the case of conventional dispensers, it is difficult to achieve a fine line (< 100 μm), since a nozzle with inner diameter of more than 100 μm should be used for dispensing relatively highly viscous ink (~ 10,000 cP).

Recently, Siddhartha Das and co-workers reported the aerosol jet printing (AJP) technique for electric circuitization and interconnects between components mounted onto different leveled surfaces with 1 mm height difference^8^. The AJP method can deposit the nanoparticles onto different level surfaces using a tilted dispense head^9^. The AJP method is also able to print fine patterns (< 10 μm) with high aspect ratio^10^. However, depending on the surface shape, complex motion control may be required, and as a result, printing speed might be slow^11^. Because of the additional units required for the generation of the atomized jet and focused carrier gas stream, the equipment for AJP is, in general, far more expensive, compared to other direct writing printing systems^12^.

In this study, we propose a new direct printing method in order to print patterns on three faces with an angle of 90° or 180°. For this purpose, we use a deflectable and stretchable jet stream from near-field electrospinning (NFES). The method has been drawing attention, because of its ability to produce fine line patterns of functional materials^3,13–16^, with relatively high printing speed of more than 100 mm/s^17,18^. So far, previous applications of NFES have focused on printing on the flat-surfaced substrate^3^. In order to use NFES for printing patterns on non-flat surface, there are several challenges that have to be overcome. First, the electrical field for jetting should be maintained constant in the presence of non-flat surfaces. Second, the printed line length should be controlled using a continuous jet process. To overcome these inherent difficulties, we modified the NFES method so that it could print lines for connecting electrode pads on both sides of a glass substrate. For conductive ink, highly viscous Ag paste ink (~ 10,000 cPs) was modified to have stretchability and flexibility by adding polymer with high molecular weight, which is described in the materials and methods. Due to the added polymer, the jet stream from the nozzle is easily deflected, and less likely to be discontinuous over the sharp edges of glass substrate.

## Method and implementation

### Direct printing for interconnecting electrodes on the opposite sides of substrates

In this work, we propose a printing method for connecting the electrodes on two opposite sides of glass substrates, which could be used in display application. Here, the conductive lines are printed on three different surfaces without complicated motion control of a nozzle with respect to the substrates. To the best of the authors’ knowledge, most of the previous methods needed complicated motion control, since printing has been performed on the substrate surface almost perpendicular to the direction of the ink being ejected (or extruded) from the nozzle.

In order to demonstrate the feasibility of our proposed method, we prepared the glass substrates having pad electrodes with the required dimensions for electrical connection in recent display applications (Table S1—Supplementary information). The pads are fabricated via inkjet printing of Ag nanoparticle ink on both sides of the substrate (Section S2—Supplementary information).

**Figure 1:**
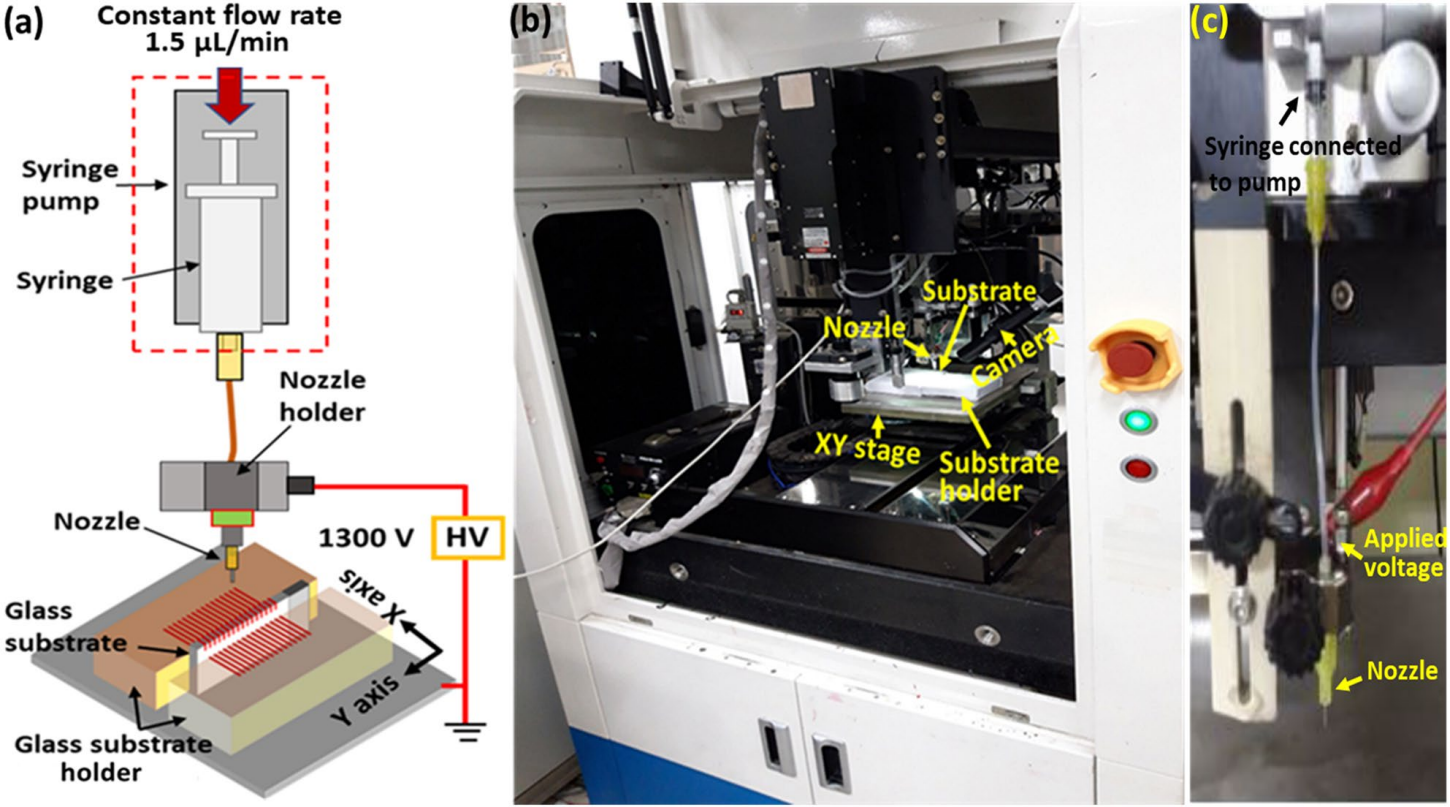
NFES printing system. (**a**) Schematic of NFES printing for connecting electrodes on both sides of glass substrate. (**b**) Photo of printing equipment. (**c**) Nozzle assembly of the equipment.

Figure 1 illustrates the implementation of the proposed printing method. The conductive ink is prepared by the mixing of commercialized Ag paste ink with polymer solution, as described in the materials and methods. Two conductive blocks with dimension of (10 cm × 10 cm × 2.0 cm) (length × width × height) hold the substrate in the vertical position, as shown in Fig. 1a,b. The vertical holding of the substrate is intended to facilitate the printing of lines over three different surfaces, without complicated motion control. For direct printing, the nozzle moves in the X-axis direction, as shown in Fig. 1a. After printing, the printed lines should be sintered, in order to achieve the proper conductivity. The required resistance of the connected lines should be less than 1.25 Ω for display application. For this purpose, different sintering methods were investigated.

### 3D surface printing using deflectable and stretchable jet from near-field electrospinning

**Figure 2:**
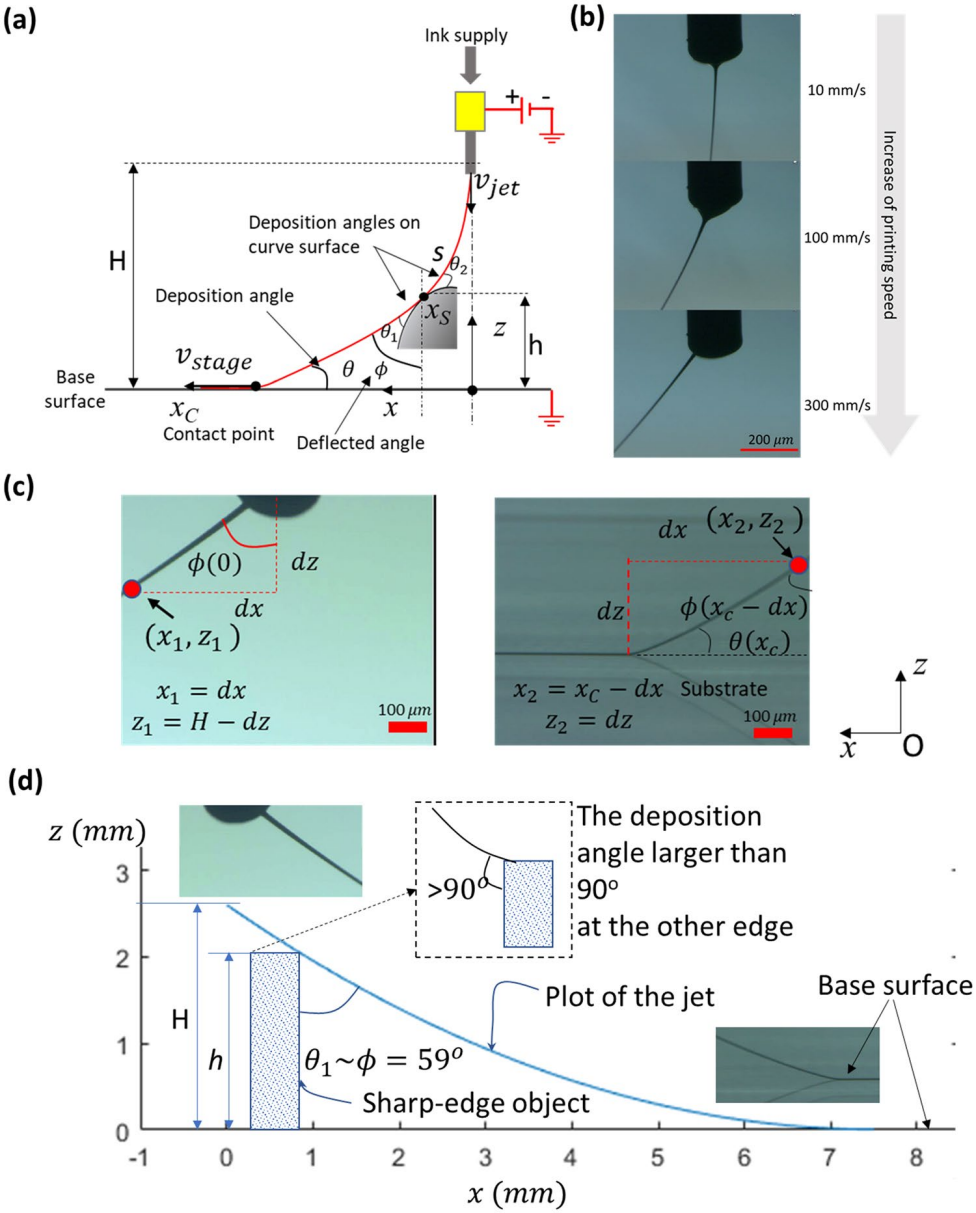
NFES jet deflection for 3D surface patterning. (**a**) NEFS jet deflection model for 3D surface printing. (**b**) Influence of printing speed on jet deflection shape. (**c**) Jet deflection images near nozzle and near contact point, which can be used for calculating $x_{C}$, and (**d**) Jet deflection plot using parabolic model in comparison to the experimental observation. Jetting parameters: Flow rate Q = 1.5 μL/min, nozzle inner diameter $d_{in} = 150 {\mu m}$, jetting voltage V = 1,300 V, Stand-off distance H = 2.6 mm.

Figure 2 illustrates the NFES jet from a nozzle under relative motion with respect to a substrate. In NFES, high voltage is applied to the nozzle, in order to produce charged threads of polymer-based ink, which is pulled down to grounded substrates. If there is relative motion of the nozzle with respect to a substrate, the ink threads, attached to the substrate and the nozzle, can be stretched to form straight printed lines, as shown in Fig. 2b,c. Assuming that the jetting process is in steady state, and the stage speed becomes the deposition rate of the thread on the substrate, the diameter, *d*, of the thread near the contact point can be written as:

1 $$ d\sim 2\sqrt {\frac{Q}{{\pi v_{stage} }}} $$

where, Q is the ink supply flowrate, and $v_{stage}$ is the relative motion speed of the nozzle with respect to the substrate (i.e. printing speed). Equation (1) shows that a finer pattern (lower *d*) could be achieved by either increasing the printing speed, $v_{stage}$, or decreasing the flow rate, Q. Note that the actual pattern width and thickness differ slightly from *d*, depending on the interaction between the ink and the substrate. In our study, we take advantage of the deflected NEFS jet for patterning on non-flat surfaces (3D objects), as shown in Fig. 2a. Note that in NFES, to ensure straight printed lines, the typical stand-off distance from the nozzle to the substrate should be less than 10 mm. For the same reason, the maximum height for the 3D object should be less than 10 mm. The 3D shape of the objects could be important for the attachment of ink thread on the surface. The deposition angles, $\theta_{1}$ and $\theta_{2}$, which are defined at the point of contact of ink thread on the 3D surface, are critical. For better 3D surface printing, these should be in the optimal range.

The spun ink thread has viscoelastic property, and is subjected to electric field, gravity, flow rate, and printing speed. In the case of high-speed printing, the ink thread has a catenary form. Bu et al. described the jet behavior using first-order differential equations to determine the form of the jet and the deflection angle, $\phi$, with respect to motion speed^19^. Then, the relationship was used for printing patterns on flat surfaces. However, it was based on numerical solutions of given parameters, and it is difficult to understand the effect of each parameter on printing.

Typical guidelines for the selection of jetting parameters can be found in Refs^17,20^. Among others, proper printing speed is one of the important parameters. The recommended printing speed should be more than 100 mm/s. Note that if the printing speed becomes very high (for example, more than 500 mm/s), the jet model from the nozzle to substrate could be simplified to the straight line.

In this study, we used the jet model based on parabolic equation, in order to explain the printing parameter effects on the non-flat surface printing, as shown in Fig. 2, as follows:

2 $$ z = a\left( {x + b} \right)^{2} $$

The two unknown coefficients, *a* and *b*, in Eq. (2) can be determined by the boundary conditions of contact point ($$x_{c}$$, 0) and the nozzle tip (0, *H*). Here, *H* is the stand-off distance. Then, we can obtain $$b = - x_{C}$$ and $$a = H/x_{C}^{2} $$. Equation (2) then becomes:

3 $$ z = H \left( {x - x_{C} } \right)^{2} /x_{C}^{2} $$

Here, we do not know $x_{C}$, and the determination of $x_{C}$ could be complex, since it depends on many parameters, such as ink flowrate, electric field, ink viscosity, ink density, and printing speed. So, $x_{C}$ has been determined by the numerical solution of differential equations, as discussed in the literature^19^. Figure 2c shows that in this study, we acquired jetting images to obtain jet locations $\left( {x_{1} ,z_{1} } \right) or \left( {x_{2} ,z_{2} } \right)$. From the measured locations, $x_{C}$ can be calculated by Eq. (3).

It is obvious that higher motion speed could increase the contact point, $x_{C}$, from the nozzle. For example, in our case of moving at 300 mm/s and the stand-off distance H = 2.6 mm, the contact point calculated from Eq. (3) was about 7.5 mm. Then, the jet model in Eq. (3) can be plotted as shown in Fig. 2d, and compared with jet images at the two end points. The estimated x_c_ was also verified by the micrometer attached to the manual linear stage, which is used for adjusting camera location to investigate the jet behavior at the nozzle and substrate. The comparison results of jet shapes from the model and images indicate that the simplified jet model can successfully predict the shape of jet deflection.

By using Eq. (3), the parameter effects on non-flat surface printing could be understood. The tangent of 90 degree minus deflection angle, tan(90-*ϕ*), along the spun thread can be calculated as a function of $$x $$ from the derivative of $$z $$ with respect to $$x$$. Then, the deflection angle, *ϕ,* can be given as:

4 $$ \phi \left( x \right) = 90^{0} - \tan^{ - 1} \left( {\frac{2H}{{x_{C}^{2} }}\left( {x - x_{C} } \right) } \right) $$

The deposition angles, $\theta_{1}$ and $\theta_{2}$, could be determined by $\phi \left( x \right)$ and the curvature of the 3D objects. Note that the smaller deposition angles provide the better deposition. If the deposition angle becomes larger than 75°, the pattern would not be well printed on the surface. In this study, the extreme printing case of a sharp object is mainly discussed, since the printing on faces with 90° or 180° (sharp edges) would be more difficult, compared to other general objects with non-sharp edges. Note that a rectangular object (glass) with sharp edges was used for printing, which has larger deposition angle, $\theta_{1}$ and $\theta_{2} ,$ compared to objects with rounded edges, as shown in Fig. 2a.

In our application, the object height, *h*, is equivalent to the extruded distance of the glass from the base of the substrate holder. In order to understand the printability on vertical faces (sharp edges), we need to estimate $\theta_{1}$, which is almost equal to $\phi$ at the point of jet contact with the object. The position of the contact, *x*_*s*_, on the glass surface can be calculated from $h = H\left( {x_{S} - x_{C} } \right)/x_{C}^{2} {\text{as}}:$

5 $$ x_{S} = x_{C} \sqrt {h/H} + x_{C} . $$

By substituting for $x$ in Eq. (4) by $x_{S}$, we can obtain the thread deposition angle, *θ*_*1*_, as follows:

6 $$\theta_{1} \approx{ }\phi = 90^{0} - \tan^{ - 1} \left( {\frac{{2\sqrt {Hh} }}{{x_{C} }}} \right)$$

Note that the deposition angle of $\theta_{2}$ is less critical compared to $\theta_{1}$, since it is related to printing on the top flat surface. Figure 2d shows the comparison of our jet model plot and measured jet deflection images. The figure shows that the calculated jet deflection behavior agrees well with the experimental observation. When we used the parameters of $x_{C} \approx 7.5\,\hbox{mm}$, $H = 2.6\,\hbox{mm}$ and $h = 2\,\hbox{mm}$, the calcuated angle, $\theta_{1}$ was about 59°, which can be verified by inserting the block image on the jet behavior plot in Fig. 2d. Based on our experimental result, deposition angle of (60–65)° can print lines of about 1 mm length on the side walls. Note that the lower deposition angle produces better printing results on the side walls. To reduce $\theta_{1}$, the object height *h* could be increased, but it has limitation. Because the jet deflection can easily be affected by coulombic repulsion caused by the deposited charged ink, the distance from the object to the nozzle, which is *H–h*, should not be too close (for example, less than 0.5 mm). Note that we used the substrate holding blocks to adjust *H* and *h*. If the height of 3D object is less than a few millimeters, the holding blocks will not be necessary. Recently, we successfully used near-field electrospinning for the interconnection of non-flat objects without supporting blocks for substrates^20^.

## Results and discussion

### Printing results

For patterning, the prepared ink was fed to a nozzle with a flow rate of 1.5 μL/min by using a syringe pump. In order to pull down the ink, a high voltage was applied to the nozzle with inner diameter of 150 μm. Here, a jetting voltage of 1300 V was applied to the nozzle, and the stand-off distance between the block surface and the nozzle tip was H = 2.6 mm. Because of the height of objects, the higher stand-off distance might be needed, compared to the case of printing patterns on flat surfaces. However, it should be noted that when the stand-off distance of more than 3 mm is required, high voltage of more than 2 kV might be needed for proper jetting^13^. The use of high voltage more than 2 kV is not recommended, because it could increase the charge repulsion from the pre-deposited lines. Considering the limitation, the extruded glass height (h) of 2 mm was used for our application.

**Figure 3:**
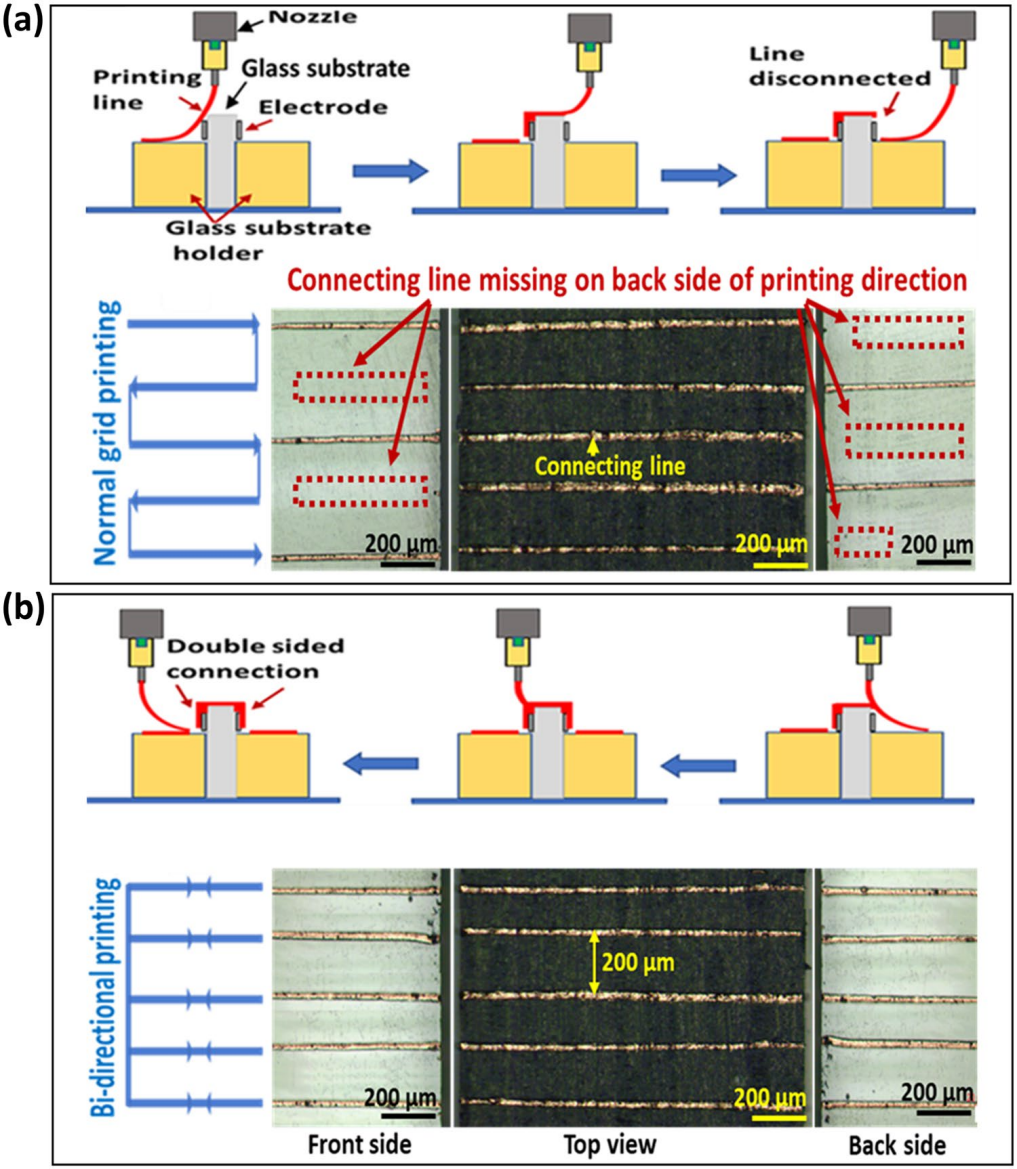
Printing methods and the typical printed results on each side of the glass. (**a**) Unidirectional printing results, and (**b**) Bi-directional printing results.

For NFES, the horizontal grids have been widely used for printing lines^17^. Figure 3 shows the printed results on each side of the extruded glass substrate along the printing direction. Figure 3a shows that in the case of using horizontal grid patterns based on unidirectional printing, we could obtain good printed results only on the first printed side of the glass. However, the other side was not printed properly. This is explained by the deposition angle. The non-printed face has larger deposition angle (> 90°), and cannot be printed properly on the other side, as shown in Fig. 2d.

To print conductive lines on both faces of the glass, we modified horizontal grid patterns such that lines can be printed bidirectionally (forward and reverse printing) at the same printing location, prior to moving to the next printing location, as shown in Fig. 3b. The bidirectional printed results verify the capabilities and effectiveness of our proposed methods for connecting electrodes on opposite surfaces.

**Figure 4:**
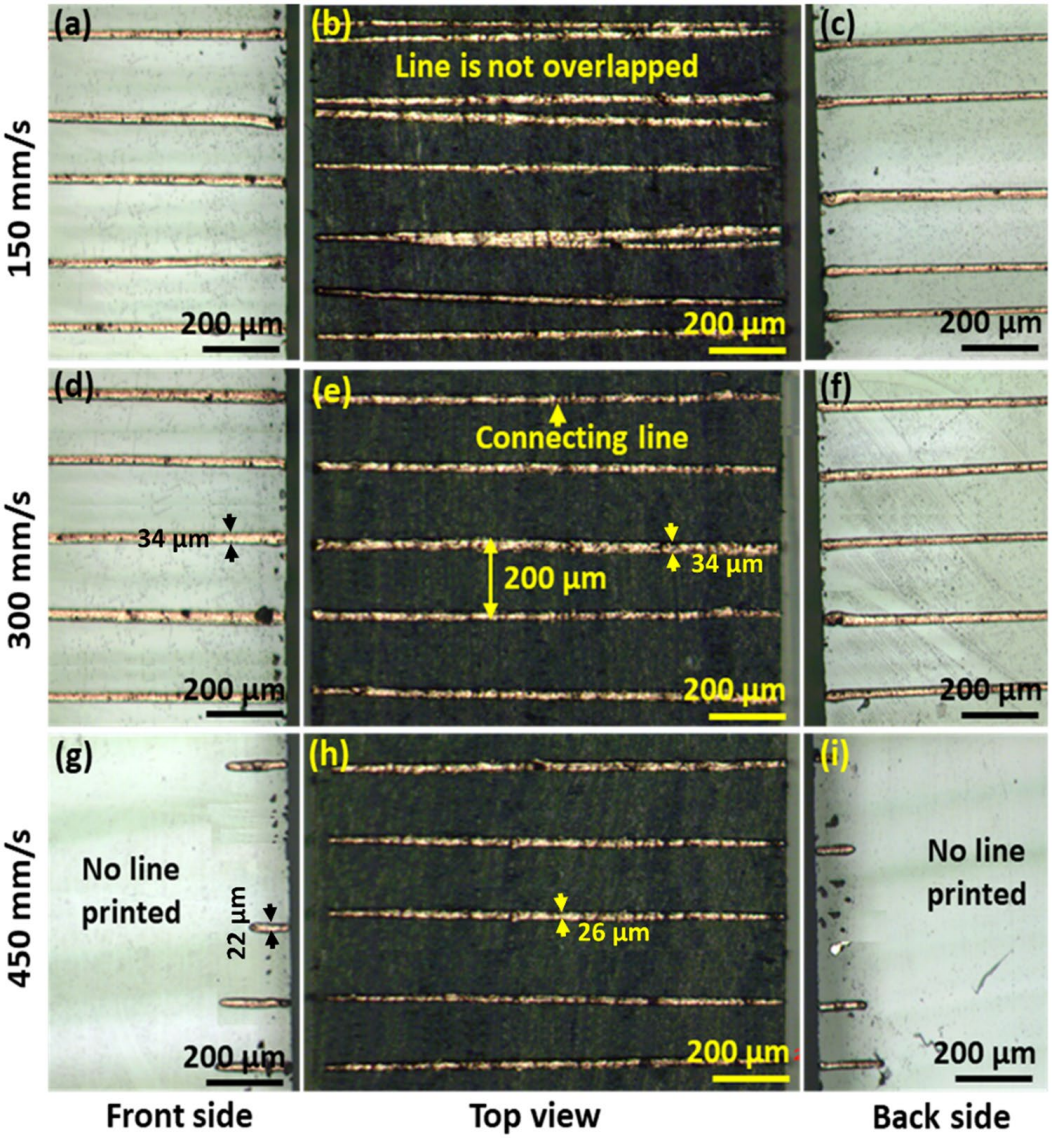
Optical microscopy images of Ag printed lines (printed line spacing of 200 μm) with respect to various printing speed. (**a**–**c**) at 150 mm/s; (**d**–**f**) at 300 mm/s; (**g**–**i**) at 450 mm/s.

The printing speed is one of the critical parameters that affect the length of printed lines and the printing accuracy on the top and side wall of glass. In industrial applications, higher printing speed is preferred, in order to increase the throughput of the process. In addition, higher speed can produce finer pattern, as shown in Eq. (1). However, higher printing speed causes larger deposition angles on the side walls, which could result in poor printed lines on side walls, as shown in Fig. 4g,i. Lower printing speed can produce smaller deposition angles for side walls patterning, as shown in Eq. (3). However, the ink thread may not be stretched enough to overcome the coulombic repulsion effects from the deposited lines on the substrate. As a result, when bidirectional printing is used, the straightness and accuracy of printed lines become poor, with improper overlapping on the top, as shown in Fig. 4b. Figure 4a–i show the printing speed effects on printed lines. From the results, the proper range of the printing speed should be used for printing on the three different surfaces. The speed should not be lower than 150 mm/s, and should not be higher than 450 mm/s. From the experimental results, the optimal printing speed was in the range (250–350) mm/s for patterning (Fig. 4d–f).

For display interconnection application, the conductive lines for printing must be aligned with respect to the electrode pads, and they should pass over the center of the pads. For this purpose, we proposed camera-assisted procedures, which are described in Section S3.1, Supplementary Information. However, the length of each printed line on the side walls could slightly differ from time to time. Excessively long printed lines could result in short circuits with other signal or power lines. In order to avoid possible short circuits and make the length of printed lines uniform, a masking-based method can be used (Refer to Section S3.2, Supplementary Information). By using procedures discussed in Section S3, we successfully demonstrated that by using the camera-assisted alignment, pads on both sides could be connected with acceptable position accuracy for pad spacing of 400 μm (Fig. S5, Supplementary information).

#### Connectivity of printed lines over sharp edges

**Figure 5:**
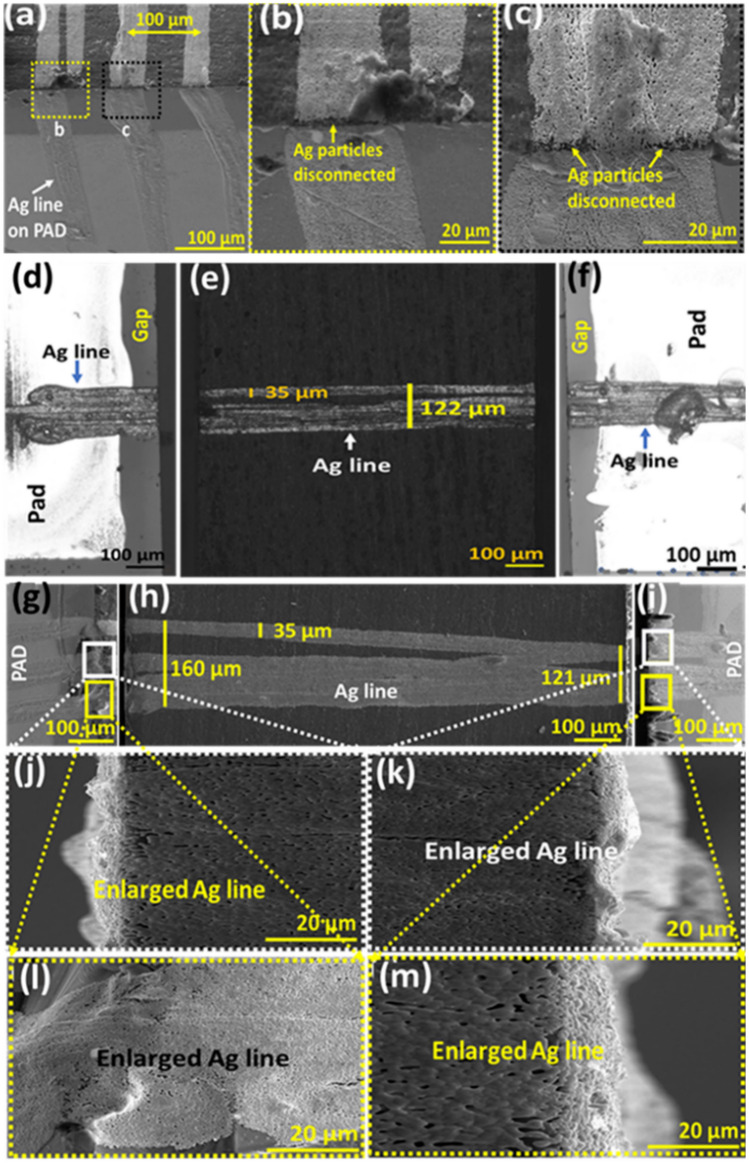
Optical microscopy and SEM images of printed lines. (**a**) Printed lines with 100 μm spacing. (**b**) Enlarged edge parts with line disconnection. (**c**) Enlarged edge parts with partial disconnection. (**d**–**f**) Optical microscopy images of multiple printed lines. (**g**–**i**) SEM images of multiple printed lines. (**j**–**m**) Enlarged image at the edges.

Figure 5a shows the printed results when the printed line spacing was set to 100 μm. The results demonstrate that the method has potential capabilities of high printing density up to 100 μm. However, it should be noted that the printed lines could be partially disconnected, due to the sharp edges, as shown in Fig. 5b,c. To solve this problem and increase the reliability of printed lines, we propose at least three times of bi-directional printing at the same location, prior to moving to the next printing locations. As a result of multiple printings, we can observe that as many as six lines are overlapped on the top side of glass, as shown in Fig. 5e,h. However, there are fewer lines on the side walls, as shown in Fig. 5d,f,g,i). The width of printed lines is about 35 μm, while overlapped multiple printed lines are within the range ± (70–80) μm, which is acceptable for most display applications. The SEM images of edge parts (boundary region) in Fig. 5g,i were enlarged as shown in Fig. 5j–m. Enlarged images in Fig. 5j–m showed that the Ag particles are well connected to form conductive lines over the edge.

**Figure 6:**
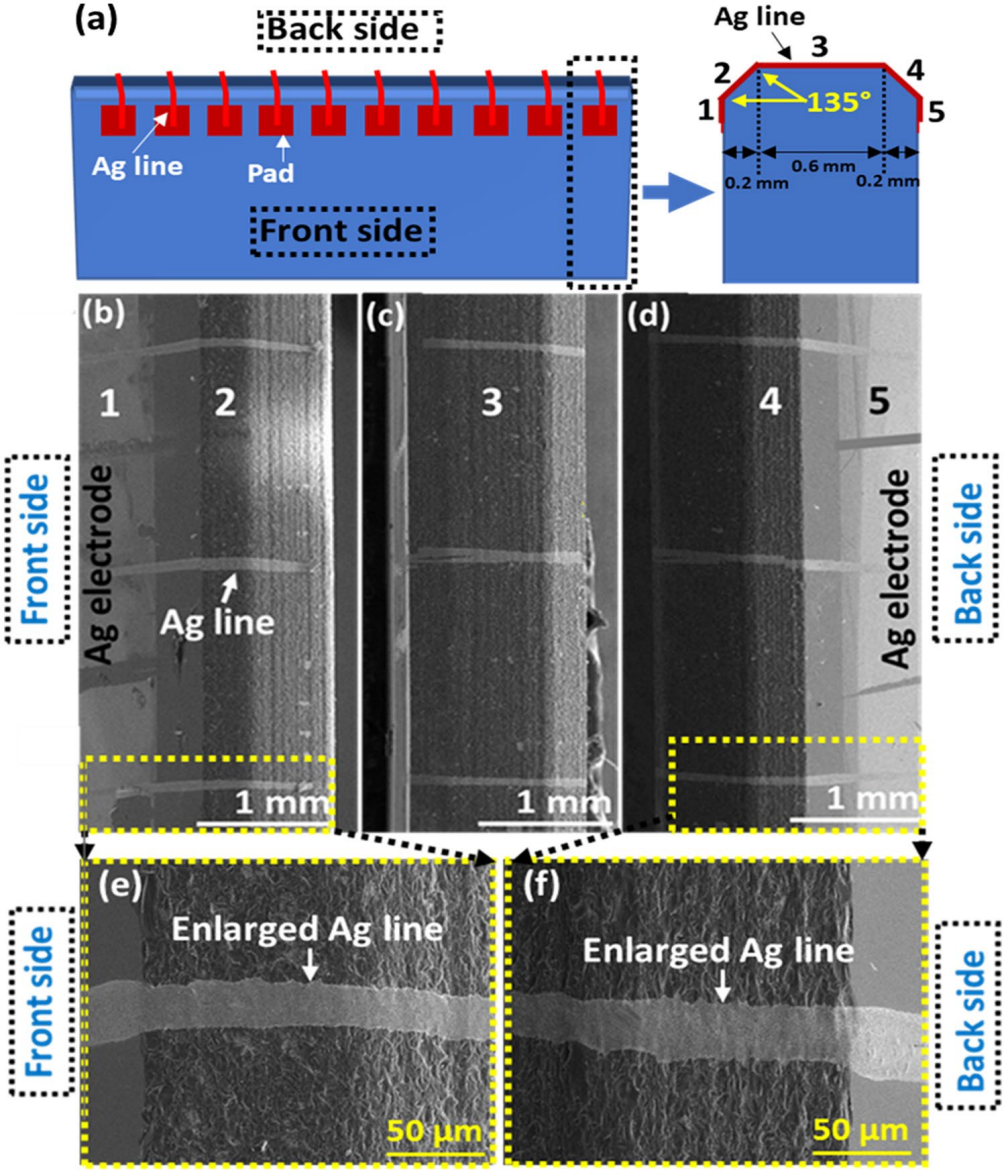
Ag lines printed over chamfered glass edges. (**a**) Schematic of printing lines between electrode pads on opposite sides. (**b**–**d**) SEM images of printed lines on chamfered glass substrate. (**e**, **f**) Enlarged images at the edges.

Multiple bi-directional printing at the same location can ensure line connection over sharp edges. On the other hand, the sharpness of the edges could be reduced for the reliability of printed patterns. For the demonstration, we used chamfered glass, as shown in Fig. 6a. Note that the chamfered dimension is very small (0.2 mm), so that the deposition angle remains almost the same ($\approx 60^{o} )$. Figure 6b–f show that only single bi-directional printing could sufficiently connect two opposite pads with a good connectivity over chamfered edges. However, industry prefers the glass without chamfered edges, because due to the extra manufacturing cost, chamfered glass substrate could be expensive.

### Sintering methods for printed lines

In this study, we report three different sintering methods of thermal, near infra-red (NIR) laser, and electrical sintering for the printed patterns over glass edges.

#### Thermal sintering

**Figure 7:**
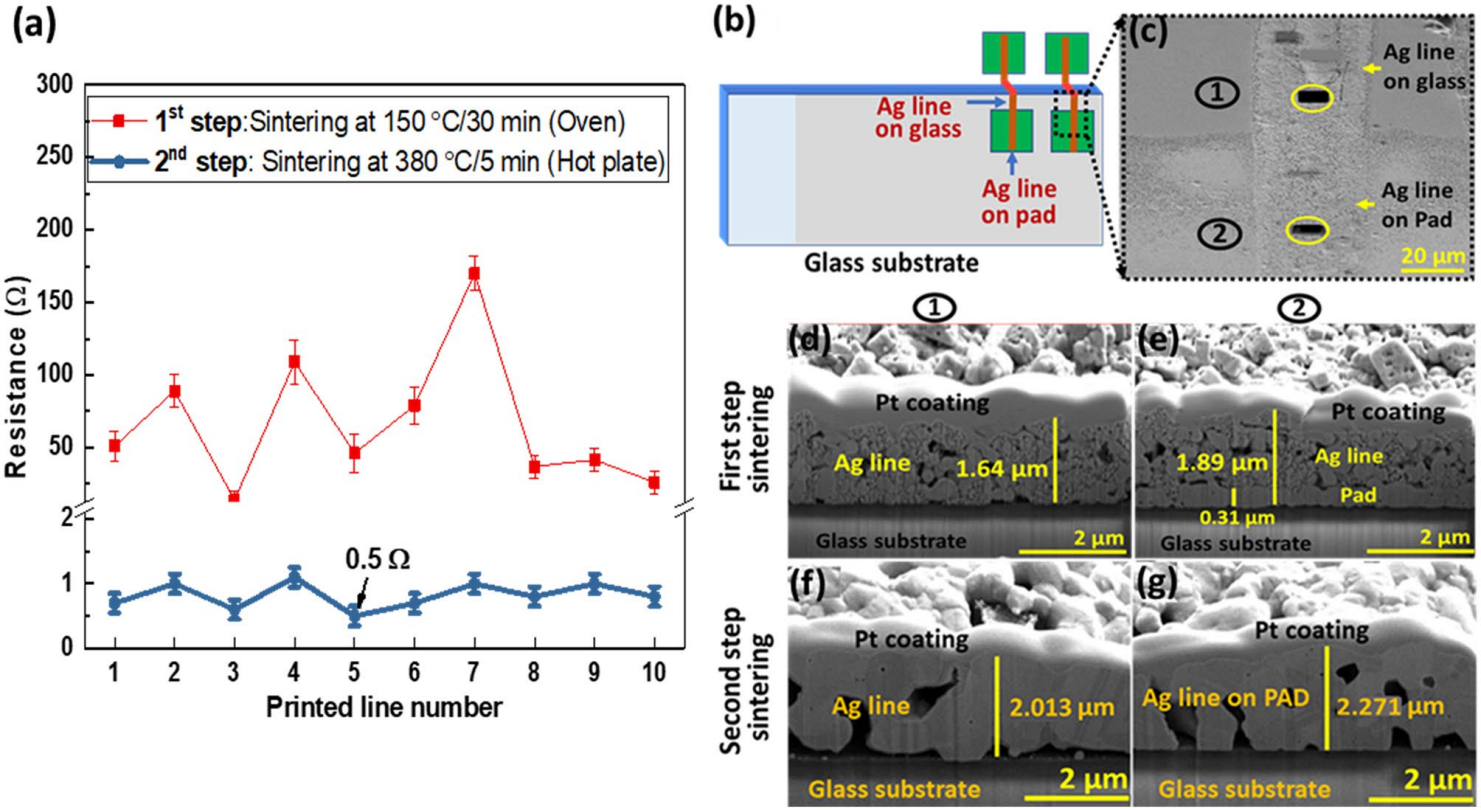
The resistance of printed lines and microscopic images of thermally sintered lines. (**a**) Measured resistances of printed lines. (**b**) Measurement locations of microscopic analysis. (**c**) FIB analysis locations. (**d**, **e**) Cross-sectional (FIB) images after first step sintering (150 °C/30 min) at locations (1) and (2). (**f**, **g**) Cross-sectional (FIB) images after second step sintering (380 °C/5 min) at locations (1) and (2).

For thermal sintering, two steps were carried out: 150 °C/30 min in convection oven, then 380 °C/5 min on hot plate. After completing the first step of sintering (150 °C/30 min), the measured resistances between the two pads were as high as hundreds of ohms, as shown in Fig. 7a. Note that thermogravimetry analysis (TGA) indicates that the first step of sintering at 150 °C would not be enough to burn organic additives in printed lines (Section S4.1, Supplementary Information). This is why we used another step of sintering at 380 °C for 5 min. Note that the second step was performed with shorter duration of time. Otherwise, the printed patterns are likely to peel off, due to burning of the adhesion promoter. Figure 7a shows that as a result of the two steps of sintering, the resistance was significantly reduced. The average resistance of 10 printed lines was 0.84 Ω, and most of the connected lines met the target resistance of 1.25 Ω.

For better understanding of the sintered lines, Fig. 7d–g show that we investigated the cross-sectional images of the printed lines from the measurement locations of Fig. 7b,c. After the first step of sintering (150 °C/30 min), it is clear from the cross-sectional images in Fig. 7d,e that the Ag particle grains are not fully connected to one another, and a lot of small pores inside the printed lines are evident. In addition, Fig. 7e shows that the printed line and pre-printed electrode pad are clearly distinguishable, which indicates that there could be contact resistance between the two layers. Here, the thicknesses of printed line and pre-printed electrode pad could be measured to be (1.64 and 0.31) μm, respectively. The thickness of the pad is only about a fifth that of the printed line, because the electrode pads are inkjet-printed using low viscosity ink (around 10 cP), which has lower Ag contents, compared to that of NFES ink.

After the second step sintering (380 °C/5 min), the FIB images shown in Fig. 7f,g reveal that the Ag particle grains in the printed lines were well connected, with significant pore annihilations. Furthermore, Fig. 7g shows that two different parts of the printed line and pad are hardly distinguishable. This indicates that the contact resistance between the pad and printed line could be negligible. However, much bigger voids are observed inside the lines. These voids are probably related to burned-out organic additives inside the printed lines. Also, they are related to the compactness of printed lines filled with Ag nanoparticles. The existence of voids in the lines could result in poor conductivity, compared to the bulk Ag. Nevertheless, Fig. 7a shows that due to the good connection of Ag particles, the resistances of printed lines were significantly reduced.

#### Localized sintering methods: near-infrared (NIR) laser sintering and electrical sintering

As an alternative to the thermal sintering method, localized sintering methods could be considered to avoid heating up the entire substrate^21–23^. In this study, we compared two different methods of near-infrared (NIR) laser sintering and electrical sintering for our application.

For NIR laser sintering, a laser source of 808 nm wavelength was used. The use of NIR laser source has the advantages of deeper light penetration (a few hundred nanometers), compared to that of near ultra-violet (NUV) and green laser (penetration depth of a dozen nanometers)^23^. Prior to laser sintering, the proper pre-baking of printed Ag patterns is an important step^24^. For pre-baking, the printed glass was placed on hot plate at 50 °C for 30 min, in order to remove the remaining solvents in the printed lines. After soft-baking, NIR laser (Class 4 laser, Losyn Yepli) was used to sinter the printed lines (Fig. S8, Supplementary Information). The focused spot size of the NIR laser was in the range (200–250) μm, which is smaller than the printed line length in the glass thickness direction (1 mm). As a result, the scanning of laser light along the printed line is required. In order to understand sintering parameter effects, the resistances of sintered lines with respect to laser powers and scanning speeds were measured as shown in Fig. S10 (Supplementary information). Based on the experimental results, we used laser power of 20 W and scanning speed of 2 mm/s for proper sintering of printed lines.

Since there are printed lines on three different faces, laser irradiation on three different surfaces might be required. However, laser scanning on three different surfaces would be inefficient, because it requires complex motion control to scan laser lights along the printed lines on each side. In order to simplify the laser sintering process, we investigated laser sintering on the single face (top side of glass), and compared it with the sintered results using laser irradiation on each printed face (three faces), as shown in Fig. S9 (Supplementary information).

**Figure 8:**
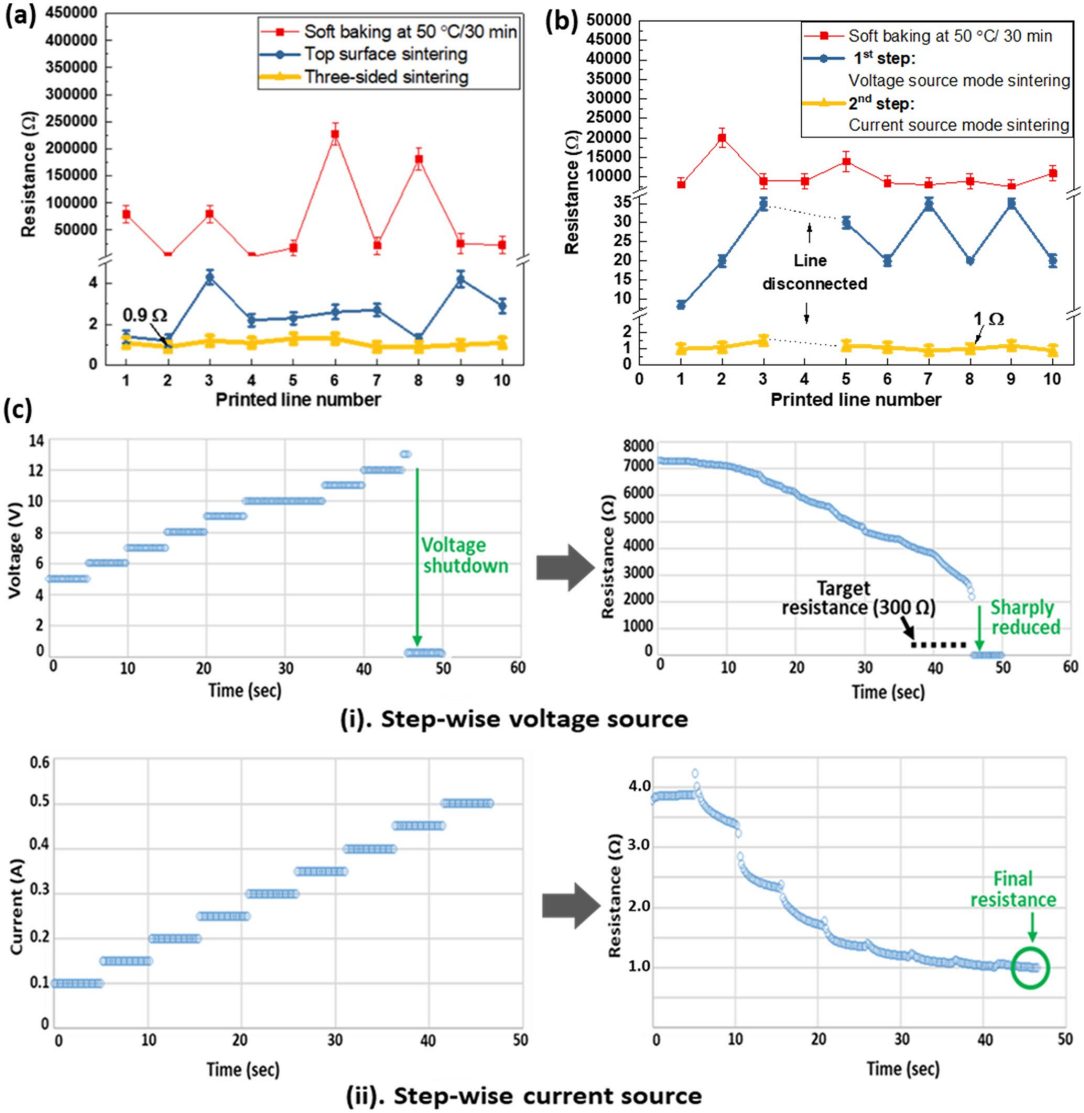
Localized sintering results. (**a**) NIR laser sintering results. (**b**) Electrical sintering results. (**c**) Electrical sintering process.

Figure 8a shows the measured resistances after single-face and three-face sintering. The figure shows that the only one-directional sintering (top surface) could reduce the resistance to an average of 3.11 Ω, which is slightly higher than 1.08 Ω of the three-face sintering. Note that the resistances of some sintered lines were as low as (1.2–1.4) Ω, which indicates that if the sintering conditions were optimized, laser irradiation on a single face could be sufficient. The possible reason is that glass has relatively low thermal conductivity (~1.0 W/mK)^25^, and generated heat on the irradiated part can be kept in the vicinity for a while. In addition, the laser light could be transmitted and scattered through the transparent glass; thereby, printed lines on the side walls could absorb light from the bottom surface of the printed lines. Note that the printed lines on the side wall are closely located at around (100–400) μm from the top edge of the glass, where the laser light is irradiated. The sintering process for only one direction could shorten and simplify the process, which can be useful for industrial applications.

Compared to other sintering methods, the electrical sintering method is straightforward, since current flow through the printed line will generate heat (joule heat) by itself, which will in turn fuse Ag nanoparticles, and burn out organic additives^26,27^. This method has advantages over other sintering methods, because it can have the inherent monitoring capability of the sintering process by measuring voltage and current in real-time. In addition, an external heat source and the heating of unnecessary parts can be avoided. The reliability of the printed lines (maximum allowable current) can be tested during the sintering process. During electrical sintering, the current density of the defected part (smaller cross-sectional part) could significantly increase, which could lead to line disconnection. As a result, only reliable printed lines could be sintered properly, and non-uniformly printed lines could be detected during the real-time monitoring.

Prior to electrical sintering, we pre-baked the printed lines by placing the substrate on hot plate at 50 °C for 30 min. After pre-baking, the initial resistance was in the range of tens of kilo-ohm, as shown in Fig. 8b. Note that the initial resistance of at least mega ohm (~ MΩ) range is required to allow initial current flow through the printed lines for joule heating. For sintering, a source meter (2400 Source meter, Keithley, USA) was used to apply electrical current through the printed lines. Two modes in the power source meter: (1) voltage source, and (2) current source, were used consecutively for electrical sintering.

First, we used a voltage source, since the initial resistance was high, and the current source mode might not work properly. The driving voltage was increased stepwise to start sintering irrespective of the initial resistance. For this purpose, the initial voltage was set to 5 V, and the voltage was gradually increased in steps of 1 V for 5 s, until the resistance reached a target value. Here, we set the target resistance as 300 Ω, in order to avoid possible damage from overcurrent. When the target resistance was reached, we shut down the driving voltage to 0 V, as shown in Fig. 8c(i). The first step of sintering using a voltage source could take about dozens of seconds (45 s in this experiment). Though the voltage shuts off, the sintering process still continues for a while due to the accumulated heat in the line, and the resistance could reduce further. As a result, after the first step of electrical sintering, the resistance could be reduced to as low as 4 Ω (initial resistance of the current mode), as shown in Fig. 8c(ii).

Secondly, the voltage source mode was switched to current source mode right after the first step of voltage-based sintering. When the resistance becomes low (under a few hundred ohm), the current source mode is a more suitable approach. Unlike the voltage source method, over-current can be avoided. However, as the resistance reduces, heat generation could reduce. In order to solve this problem, the driving current was increased stepwise. For the second step of sintering, the initial current was set to 0.1 A, and the driving current was increased with increasing step of 0.1 A for 5 s, until the resistance no longer decreases, as shown in Fig. 8c(ii). In this second step of current mode, we did not set a specific target resistance, so that we could obtain the lowest possible resistance. For example, we stopped the second step of sintering when the resistance no longer decreased. Here, we obtained the final resistance of around 1 Ω when the length of the printed line was 1.2 mm. Figure 8b provides in detail the typical resistances after two consecutive steps of the electrical sintering process. The electrical sintering method can detect the defective lines. For example, after pre-baking, all the lines in Fig. 8b show initial resistances of less than 20 kΩ. This indicates that all lines are connected. However, there was a disconnection case during the first step of the sintering process, as shown in Fig. 8b, line number 4. The microscopic images of disconnected lines could show the over-burn spots due to the over-current (Fig. S12, Supplementary information).

We note that the final resistance of sintered lines could be similar, regardless of sintering methods, as shown in Figs. 7 and 8. Comparison of the cross-sectional microscopic images of the printed lines (Sect. 4.5, Supplementary Information) indicates that if the conditions are optimized, the sintered results could be similar. Optimization of the sintering methods is beyond the scope of the work. For the selection of sintering methods, practical requirements, such as takt time, monitoring capability, and reliability, could be important factors.

## Concluding remarks

In this study, we discussed how to print conductive lines on three-dimensional surfaces, including sharp edges, to connect the electrodes on both sides of the glass. Our proposed method does not require complex 3D motion control of the nozzle, since surfaces that are both perpendicular and parallel to the jetting direction can be printed.

In conventional NFES printing, it is necessary to create electrostatic force that pulls the jet down to the substrate. Therefore, the ground voltage should be connected to the other side of the printing surface. However, in this method, ground voltage was connected to the two blocks of the substrate holder, which does not produce direct attractive force for ink deposition on the glass surface. Instead of using the direct attractive force of jet, we used continuous jet, which was maintained while passing over a 1 mm thick glass. We demonstrated that when using a relatively fast printing speed, the continuous jet was uninterrupted during the printing of sidewalls and the top of the glass.

An important issue of using liquid materials to deposit over sharp edges is the reliability of the connected lines. In order to ensure the connectivity, bi-directional printing was repeated three times at a target location. The accuracy of multiple printed lines was within ± (70–80) μm, which is acceptable for recent display applications. When the resistance between the pads located on both sides of glass was measured, the average resistance of connective lines was about 1 Ω.

We believe that our proposed printing and sintering method can be extended to various applications, such as substrates with 3D or 2.5D surfaces with sharp edges. Due to the electronic components mounted on the surface, there may be locally stepped objects on the substrate. The height of the step can be more than 500 times (in the millimeter range), compared to the pattern thickness (in the micrometer range). Such substrates should be considered 3D or 2.5D surfaces, and our proposed direct printing method can also be effectively used to connect electrical components having different heights.

## Materials and methods

### Substrate preparation

For experimental demonstration, glass slides of 1 mm thickness (Microscope slides, Marienfeld Superior, Germany) were purchased. For comparison, chamfered glass substrates were also used, which were donated by Samsung Electronics, South Korea. Then, conductive pads were inkjet-printed on both sides of the glass substrates (Section S2, Supplementary Information). For inkjet ink, Ag nanoparticle ink (Silverjet DGP 40LT-15C, ANP, South Korea), which contains 30.63 wt% of Ag content, was used. Here, two different size of electrode pads with dimension of (0.3 mm × 0.28 mm) and (1 mm × 1 mm) were inkjet-printed. The printed lines were to connect both pads on opposite side, and the resistance of printed lines could then be measured by probing the two pads. To demonstrate electrical sintering, we printed the pads with different dimension on each side: (1 mm × 1 mm) on the front side, and (3 mm × 1 mm) on the back side of the glass. After printing, the printed pads were sintered in convection oven for 30 min at 150 °C.

### NFES ink preparation

We purchased Ag paste ink (ES-INK, NPK, South Korea), which contains 85.5 wt% of Ag content, with typical viscosity of 11,200 cP. To enhance the stretchability of the conductive ink, Ag paste ink was mixed with polymer solution. Polymer solution was prepared by dissolving 0.3 gm of polyethylene oxide (PEO), (M_v_ = 400,000) (MFCD00081839, Sigma-Aldrich, USA) in 11 gm of mixture solvents composed of ethanol (8.8 gm) and deionized (DI) water (2.2 gm). In order to obtain fully dissolved polymer solution, the mixture was stirred for 12 h by magnetic stirrer (SP131320-33, Thermo Scientific, China). As a final step, Ag paste ink of 10 gm and the polymer solution of 2 gm were mixed for 10 min by vortex mixer (VM-96E, Jeio Tech, South Korea). The whole mixture process was carried out at room temperature of 26 °C with relative humidity of 40%. After preparation, the viscosity of the NFES ink was measured at 26 °C by rheometer (Brookfield DV-III ultra, Brookfield Engineering Lab., Inc., USA). The ink has viscosity ranging (6400 to 490) cP depending on the shear rate (spindle speed) (Section S4.1, Supplementary Information).

### Printing method

The prepared NFES ink was fed to a syringe needle having an inner diameter of 150 μm with a flow rate of 1.5 μL/min, as shown in Fig. 1a. To supply the ink with constant flow rate, a syringe pump from Nanojet (Nanojet 47963, Chemyx Inc. USA) was used. Then, the high-voltage power supply (SHV30R, ConverTech, South Korea) was connected to the needle holder. Glass substrate was vertically positioned with the printing part extruded about 2 mm from the substrate holder. To produce an electrical field for the Taylor cone jet, the nozzle and substrate holders were connected to 1300 V and ground, respectively, which pulls down the charged ink from the nozzle to the substrate holder, as shown in Fig. 1. The laboratory-developed NFES printing system shown in Fig. 1b,c was used for the printing experiments.

To obtain a uniform printed result, we performed idling printing for more than 30 min on a paper substrate, in order to ensure steady state jetting prior to printing. While idling printing, printing parameters, such as flow rate (1.5 μL/min), stand-off distance (nozzle tip to substrate holder distance, H) (2.6 mm), and high voltage (1.3 kV) applied to the nozzle part, were used to produce the desired steady-state jet stream. To obtain straight printed patterns along the printing direction, the printing speed was set to 300 mm/s. The printing location was aligned with respect to the electrode pads by the use of camera assistance procedures (Section S3.1, Supplementary Information). The printing location was aligned with respect to the electrode pads. To increase the reliability of connection at the sharp edges of glass, bi-directional printing was repeated three times at the target location, prior to moving to the next printing location.

### Sintering of printed line

After printing, the printed lines should be thermally sintered to obtain sufficient conductivity. The thermal sintering was carried out in two steps based on thermogravimetric analysis of the ink (Fig. S7, Supplementary information): 150 °C in convection oven (Vo-27, HYSC-Hanyang Scientific Equipment, South Korea) for 30 min, and then on hot plate (SP131320-33, Thermo Scientific, China) for 5 min at 380 °C. Other alternative sintering methods, such as NIR laser sintering and electrical sintering, were investigated, in order to avoid the heating of the entire substrate.

### Characterization of sintered line

After sintering, the resistance (R) of printed lines was measured by 4-wire connection method by using multimeter (GDM-8341, GW Instek, China) and source meter (2400 Source meter, Keithley, USA). The current source of 100 μA was used to drive current through the printed lines from the two pads. Then, voltage was measured using multi-meter at the two pads. The resistance was calculated by measured voltage divided by driving current. Note that when the directly measured resistance (2 W method) was used, the effect of contact resistance was about (0.1–0.01) Ω, depending on probe contact.

The microscopic structure of connected lines was characterized using scanning electron microscopy (SEM) (Mira 2, Tescan, Czech Republic), and optical microscopy (XTCam-D310M, Mitutoyo Measuring Microscope, Japan). In addition, the cross-sectional microstructure image was taken using focused ion beam (FIB) system (Lyra 3, Tescan, Czech Republic).

## Supplementary information

Supplementary Information 1.

Supplementary Video 1.
