# Supplementary material for: Three-dimensional surface printing method for interconnecting electrodes on opposite sides of substrates
Source: Sci Rep. 2020 Oct 29;10:18645. doi: 10.1038/s41598-020-75556-x (PMC7596720; doi:10.1038/s41598-020-75556-x)
Supplement: Supplementary file 1 — Supplementary Information 1. [file 41598_2020_75556_MOESM1_ESM.docx]

Supplementary Information

Three-dimensional surface printing method for interconnecting electrodes on opposite sides of substrates

**Md. Khalilur Rahman^+^, Seong-jun Kim^+^, Thanh Huy Phung, Jin-Sol Lee, Jaeryul Yu, Kye-Si Kwon***

^+^ Both authors contributed equally to this work.

*Corresponding author: **Prof. Kye-Si Kwon**, **Ph.D.** Department of Mechanical Engineering,

Soonchunhyang University, 22, Soonchunhyang-ro, Asan city,

Chungnam, 31538, South Korea

Email: [kskwon@sch.ac.kr](mailto:kskwon@sch.ac.kr)

Website: <http://inkjet.sch.ac.kr/>

**This PDF file includes:**

Supplementary Information Text

Figures S1 to S14

Tables S1 to S2

Other Supplementary data for this manuscript include the following:

Movie S1

**Supplementary Information Text**

*S1. Experiment overview*

Figure S1 shows the schematics of the entire experimental procedure in this study, including the printing and sintering processes.


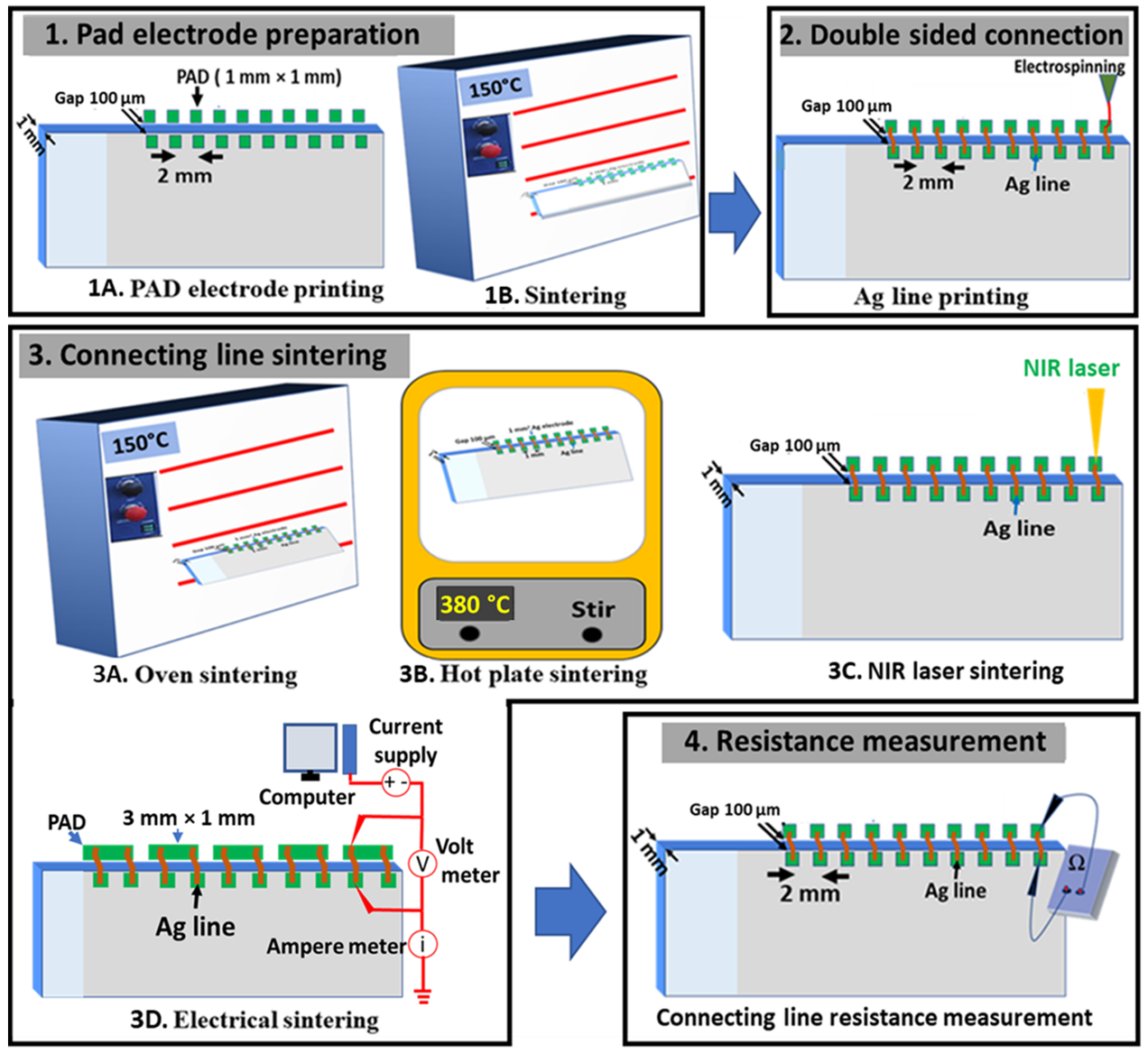


**Figure S1. Experimental procedures.**

*S2. Pad electrode preparation*

Recent displays have shown a very narrow boundary for the bezel, where electrode pads are located for various connection purpose, such as external power, as well as signal connection from the other side of substrate. To demonstrate our proposed method, we used glass slide with thickness of 1 mm, without loss of generality. The schematic in Fig. S2(d) illustrates the electrical line connection between two pads on the opposite side of the glass. Note that the electrode pads are located equally spaced in the bezel area. Table S1 and Fig. S2(a) show our target dimensions, which have been required in recent display applications. In this study, the electrode pads were prepared via inkjet-printing using Ag nanoparticle ink (Silverjet DGP 40LT-15C, ANP, South Korea).

The electrode pads were used for multi-meter probing, in order to evaluate the printed line resistance. However, it is difficult to measure the resistance when the pads are small in dimension, as shown in Table S1. For easy measurement, substrates with larger sized pads are also prepared, as shown in Fig. S2(b). In addition, the pads are also used for applying electrical current for electrical sintering purpose, which is discussed in the main text. For electrical sintering purpose, we printed the pads with different dimension on both sides: (1 mm × 1 mm) on the front side; (3 mm × 1 mm) on the back side of glass, as shown in Fig. S2(c) and Table S2.


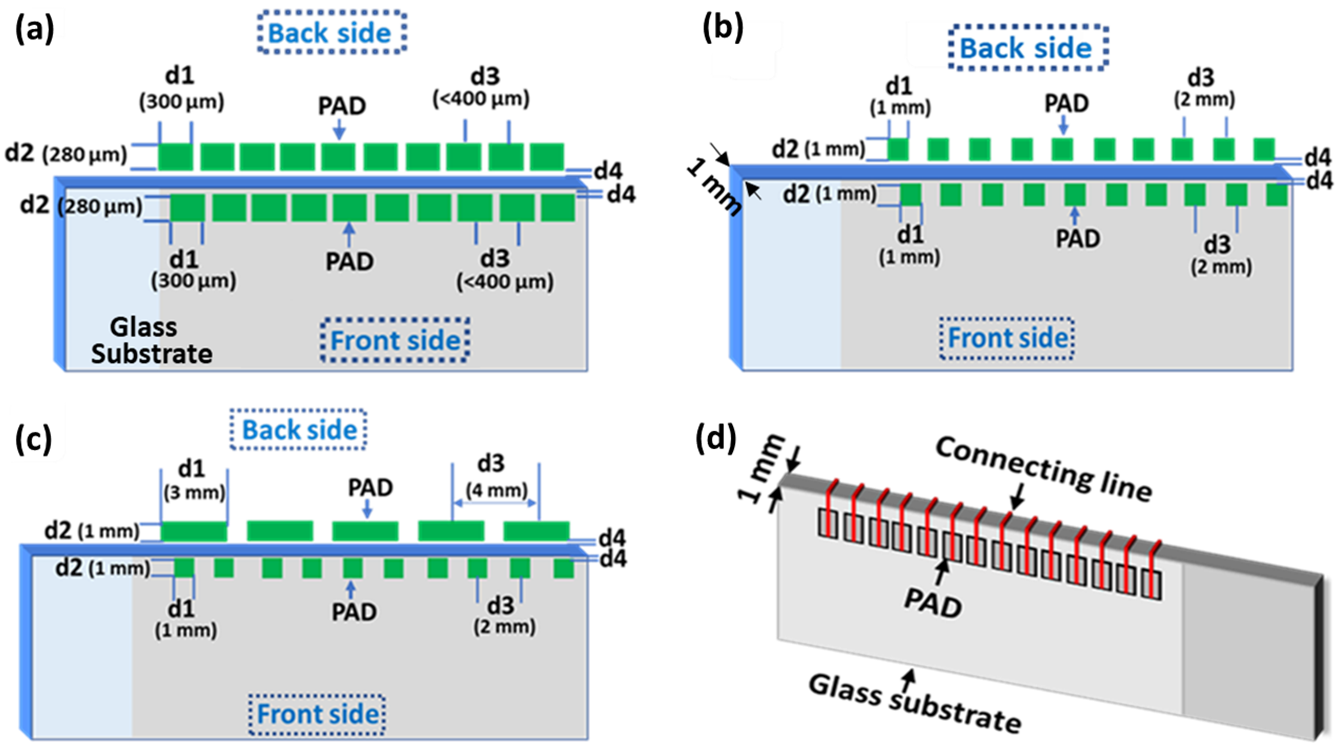


**Figure S2. Layout and dimensional parameters for electrode pads.** (**a)** Dimension of electrode pads for recent display applications. (**b)** Electrode pads for easy resistance measurement of printed line. (**c)** Electrode pads for easy electrical sintering. (**d)** Schematic of the double-sided connection requirement.

**Table S1.** Dimension parameters for electrode pads used in recent display application.

| Parameter | Dimension | Remark | |
| --- | --- | --- | --- |
| d1 | 300 μm | Pad width | Same size on front and back sides of the glass |
| d2 | 280 μm | Pad length |  |
| d3 | < 400 μm | Pad spacing (pad density) |  |
| d4 | (50–100) μm | Distance from the edge end  of glass to the pad edge |  |
| Resistance | <1.25 Ω | Double sided connecting line resistance requirement | |
| Thickness of glass | (0.75–1) mm | For experiment, glass with thickness of 1 mm was used. | |

**Table S2.** Dimension parameters for larger sized electrode pads for easy resistance measurement and electrical sintering purpose.

| Sintering method | Parameter | Dimension | Remark | |
| --- | --- | --- | --- | --- |
| Thermal and NIR laser sintering | d1 | 1 mm | Pad width | Same size on front and back sides of the glass |
|  | d2 | 1 mm | Pad length |  |
|  | d3 | 2 mm | Pad spacing |  |
|  | d4 | 100 μm | Distance from the edge end of glass to the pad edge |  |
| Electrical sintering | d1 | 1 mm | Pad width (Front side) | |
|  |  | 3 mm | Pad width (Back side) | |
|  | d2 | 1 mm | Pad length (Same on front and back sides) | |
|  | d3 | 2 mm | Pad spacing (Front side) | |
|  |  | 4 mm | Pad spacing (Back side) | |
|  | d4 | 100 μm | Distance from the edge end of glass to the pad edge (Same size on front and back sides) | |

Electrode pads were inkjet-printed via our laboratory developed inkjet printing system shown in Fig. S3. The printing procedures were as follows: 1) printing of pads on one side; 2) soft-baking at 50 °C for 10 min on hot plate; 3) printing of pads on other side of the glass; here, the printing location of pads was aligned with the pre-printed pads on the other side. 4) The printed glass was placed in convection oven at 150 °C for 30 min for sintering of the printed pads on both sides.


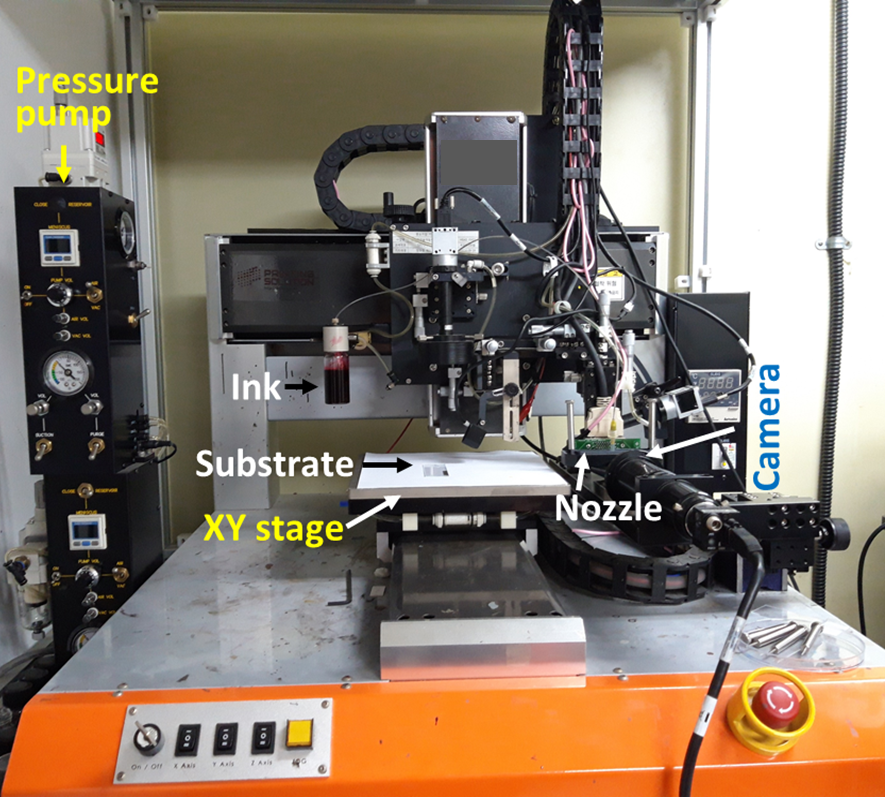


**Figure S3. Laboratory developed inkjet printing equipment.**

*S3. NFES printing process*

*S3.1* *Experimental procedures for conductive line printing over glass edges using NFES*

Figure 1 of the main text shows the schematics and a photo, respectively, of the experimental setup to connect electrode pads on the opposite sides. To print conductive lines for connecting pads on both side walls, the height of the conductive blocks (substrate holder) should be adjusted so that a small part of the glass can be extruded, as shown in Figs. S4(a–d). In this work, the distance between the nozzle tip and the substrate holder was set to (H = 2.6 mm), while the distance between the needle tip and the glass top (edge) was set to h_1_ = 0.6 mm, as shown in Figs. S4(c,d).

For printing alignment purpose, we used magnified images of pads acquired by charge-coupled device (CCD) camera (acA-1300-60gc) with 8× magnification lenses (MML8-ST653). Based on the acquired images, we used the following procedures for printing alignment, as shown in Fig. S4:

1. Set up printing parameters, such as the number of printing lines, origin (starting) XY position, printing speed, and printing locations.
2. Print lines on the two blocks covered by papers, without inserting the glass substrate. The papers on the conductive blocks can prevent electrical sparks caused by direct contact of charged ink with the grounded substrate holder. Also, the printed lines on the paper were used as alignment marks for inserting the glass substrate.
3. Insert glass substrate between the two blocks, so that the center of pads is aligned with the printed lines on the papers. For precision alignment, side-view camera image is used to view the printed line on the paper, as shown in Fig. S4(c). If the printed line is not located in the center of the acquired image, the motorized stage is used to adjust the camera location, so that the line can appear in the center of the acquired image. Then, the camera position moves up in the z stage, in order to view the pad location on the glass. Adjust the glass substrate location to locate the center of the pad at the center of the camera image. As a result of the two steps of centering, the pad center is located in the printing path, and printed lines are connected on both electrode pads, as shown in Fig. S4(e).

As a result of the alignment procedures, printed lines could be aligned to the center of pads, and printed lines will then pass over the center of pads, as shown in Fig. S4(e). Note that the length of every printed line could differ, as shown in Fig. S4(e), and the excessive length beyond the lower end of the pad could result in short-circuits with other signal lines. Section S3.2 discusses the method of controlling the length of printed lines to avoid such kinds of possible short circuits.


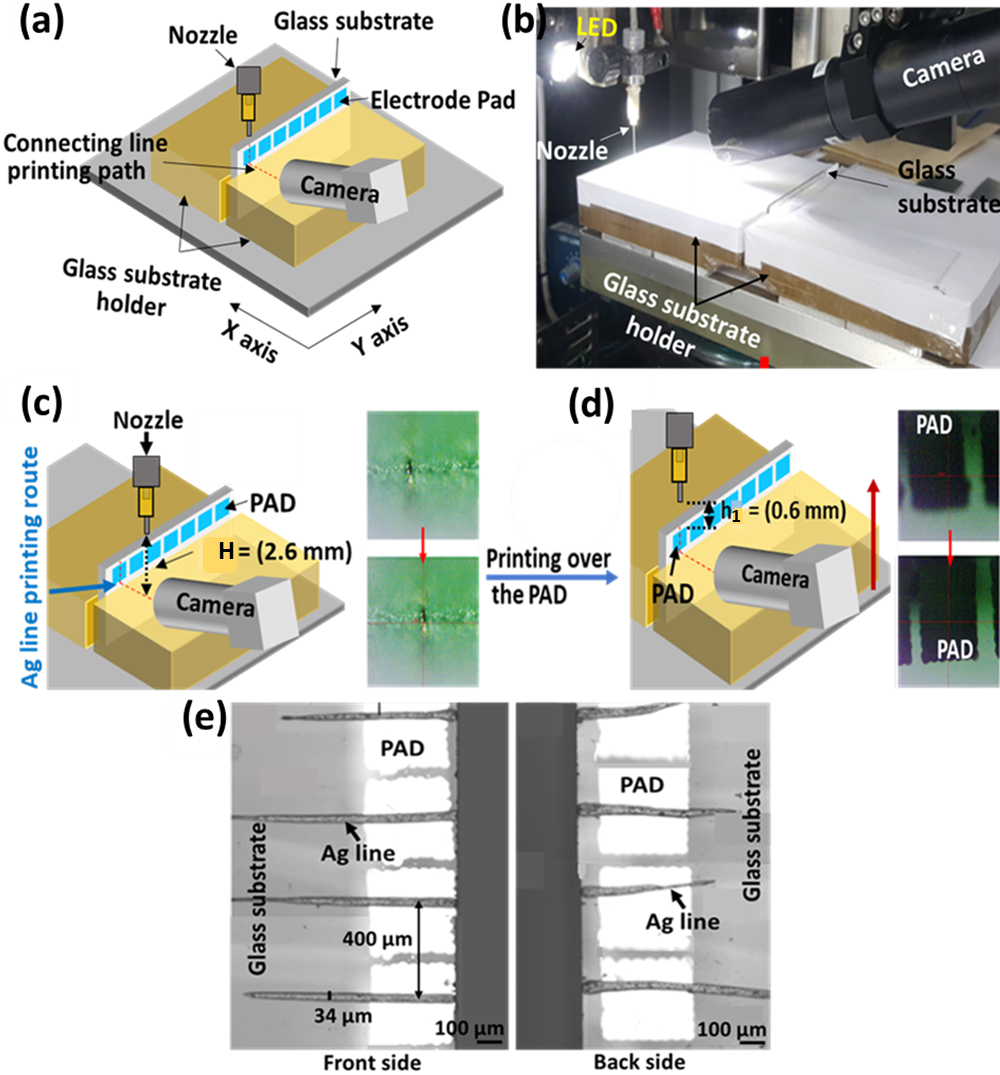


**Figure S4. Procedures for aligning printing location with respect to electrode pads.** (**a)** Schematic of the experimental setup. (**b)** Photo of the experimental set-up. (**c)** Camera alignment with respect to printed line on paper. (**d)** Adjustment of pad center location with respect to camera center. (**e)** Printed patterns on the target location of electrode pads (Front and back side of glass).

*S3.2. A masking method for uniform line length*

As discussed in the main text, the critical parameter for deciding line length is printing speed. However, even though the same printing parameters are used, the length of printed lines could vary slightly. In order to achieve uniform and consistent printing length, we propose the use of a mask to trim the printed line, as shown in Fig. S5. For masking purpose, we used post-it paper (Sticky note, Printec, South Korea), because it can easily be attached to glass substrate, as shown in Fig. S5(a). On the other hand, it can be easily removed without damaging the pads, as well as other printed patterns. In addition, printed lines should be attached well to the electrode pads in the presence of thick masking paper. Note that the thickness of masking paper would be 100 μm, which is significantly thicker than that of the printed line (~2 μm).


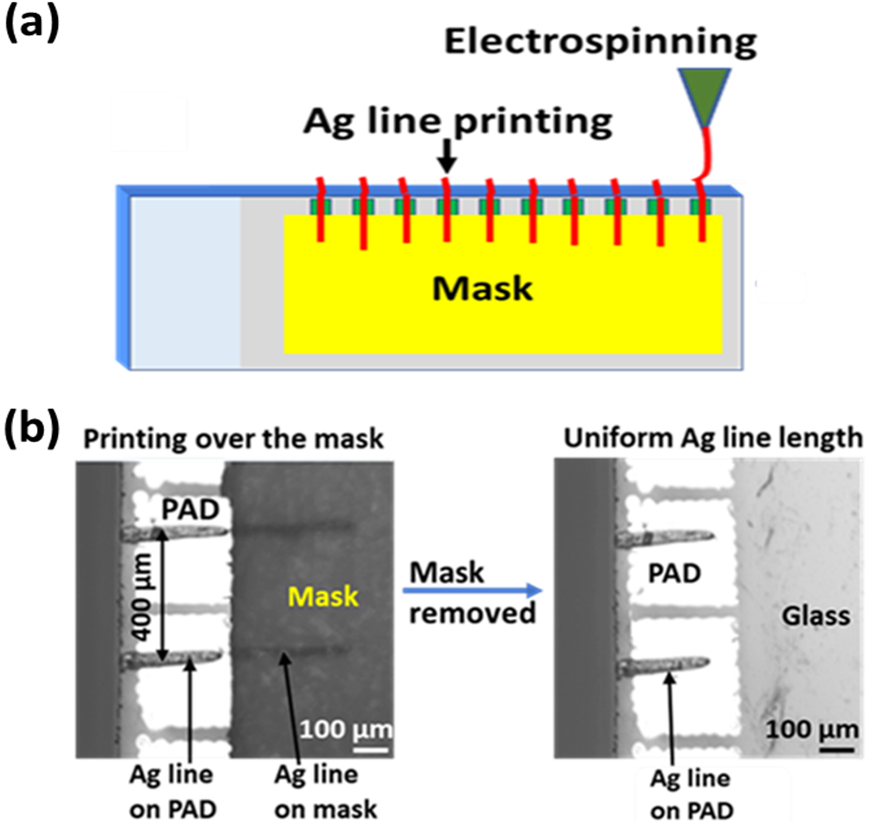


**Figure S5. Procedures for masking method for limiting printed line length and the typical results**. (**a)** Masking method to obtain uniform printed line length on side walls. (**b)** Optical microscopy images of typical printed results, before and after removing mask.

To investigate any issues of mask process, we performed printing with mask paper on the substrate, as shown in Fig. S5(a). The typical results in Fig. S5(b) show that by using the masking method, trimmed printed lines could be obtained without any damage of pads or printed lines.

*S4. Sintering methods for the printed lines*

*S4.1. NFES ink characterization*

The viscosity of near field electrospinning (NFES) ink was measured at 26 °C by rheometer (Brookfield DV-III ultra, Brookfield Engineering Lab., Inc., USA). Figure S6 shows that the ink displays non-Newtonian fluid behavior with decrease in viscosity under shear strain.


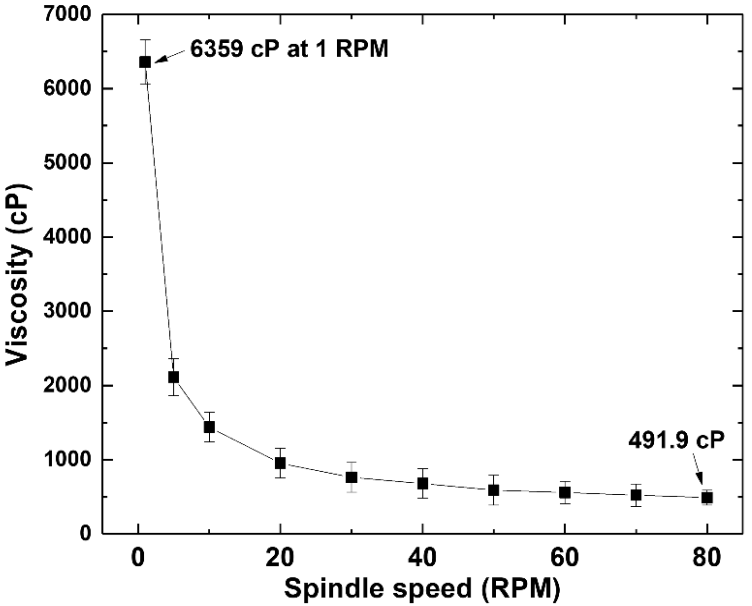


**Figure S6. Viscosity of NFES ink.**

To determine the proper sintering condition, it is important to understand the thermal behavior of the prepared NFES ink. For this purpose, we investigated the thermal properties of the ink using thermogravimetric analysis (TGA) and differential scanning calorimetry (DSC), as shown in Fig. S7. For the measurement, the ink sample was heated from (30 to 800) °C at a ramp rate of 10 °C /min under nitrogen atmosphere. Figure S7 shows that at around 38 °C, the rate of weight loss started to increase rapidly, which showed good consistency with the DSC results. At 50 °C, the DSC result shows rapid endothermic response, where one of the solvents in the NFES ink evaporated. From TGA analysis, we observed that at 135 °C, most of the solvents were evaporated; at 380 °C, the organic additive added to the ink started to decompose; and at 405 °C, was completely decomposed. The total loss of weight was about 24 %, which corresponds to Ag contents of 76 wt.%. in the prepared NFES ink.


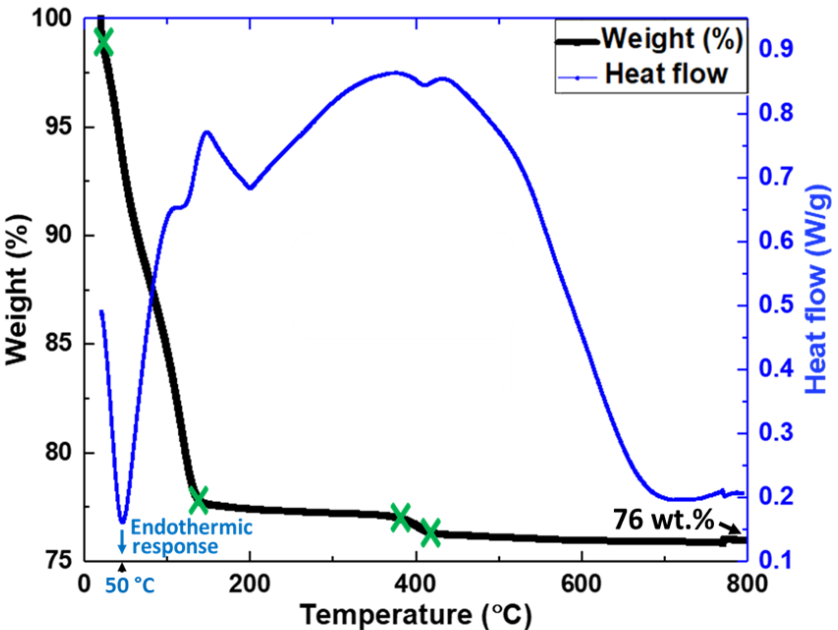


**Figure S7. TGA analysis and DSC result of the prepared NFES ink.**

The printed line using Ag nanoparticle ink should be sintered properly, in order to fuse the nanoparticles for the line conductivity. For comparison, three different methods of thermal, NIR laser, and electrical sintering were performed, since each method has advantages and disadvantages.

*S4.2.* *Near Infrared (NIR) laser sintering*

The laser sintering method is one of the localized sintering methods, which can affect the irradiated parts only. The power of NIR laser (Class 4 laser, Losyn Yepli) and scanning speed were controlled, and the laser light could be focused on the target location, by using the laboratory developed equipment shown in Fig. S8. Figure S9 shows the laser irradiation processes on the top side and three different surfaces of printed line. Figure S10 shows the measured resistance of three face-sintered printed lines with respect to laser power and scanning speed. Based on scanning experiment, the sintering condition of laser power of 20 W and scanning speed of 2 mm/s was selected.


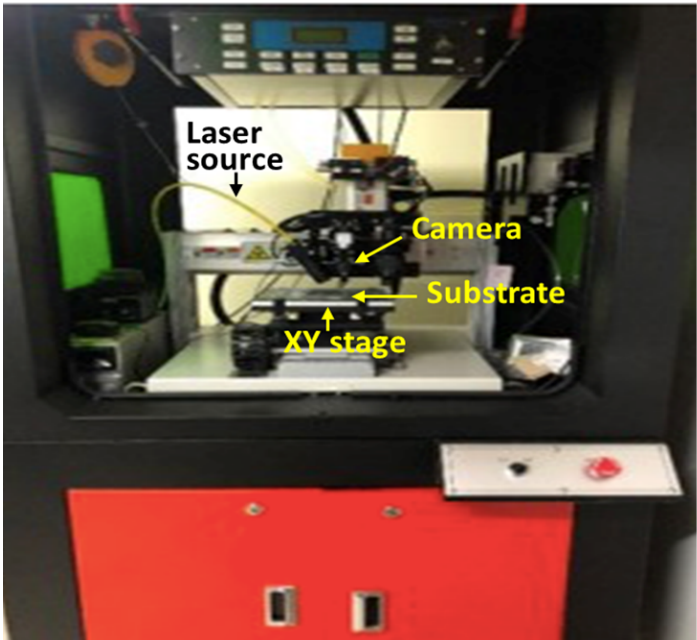


**Figure S8. Laboratory developed NIR laser sintering equipment.**

**
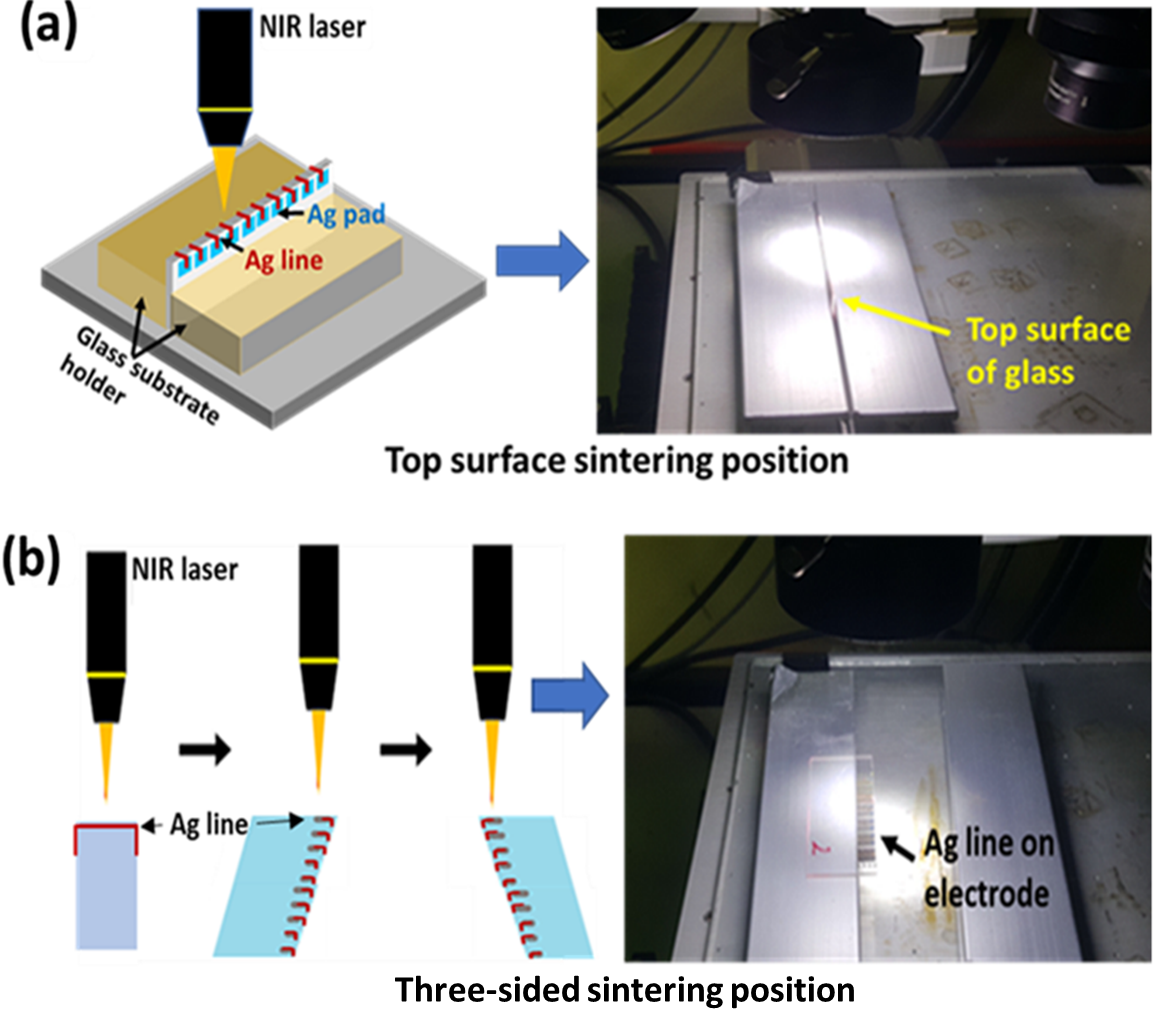
**

**Figure S9. Schematic and photo of NIR laser sintering method for printed lines**. (**a)** Top surface sintering of printed line. (**b)** Three-face sintering of printed line.

*
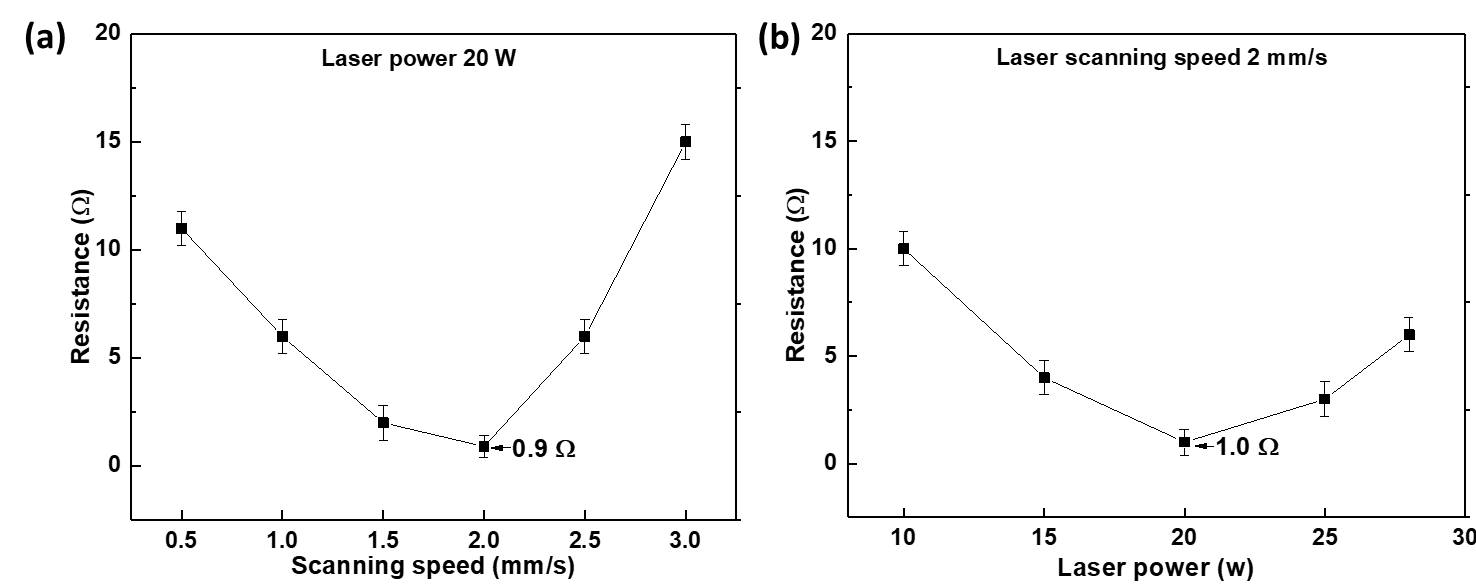
*

**Figure S10. NIR laser sintering parameter effects.** (**a)** Resistance of the printed lines with respect to laser scanning speed. (**b)** Resistance of the printed lines with respect to NIR laser power.

*S4.3 Electrical sintering*

Prior to electrical sintering, we pre-baked the printed lines by placing the substrate on hot plate at 50 °C for 30 min to obtain the initial resistance of less than mega ohm (~MΩ). Then the source meter (2400 Source meter, Keithley, USA) was used to apply electrical current through the printed lines. LabVIEW (National Instrument, USA) based software was developed to control the source meter via PC communication, as shown in Fig. S11(d). For proper sintering results, two steps of voltage and current source modes are used. To demonstrate our proposed method, we need to apply electrical power (voltage or current) to two electrode pads on opposite sides of the glass. For this purpose, the substrate needs to be vertically positioned for probe contact on both pads, as shown in Fig. S11(b), probe connection method 1. However, probing on both sides of glass might not be easy in practice. For easier demonstration, electrode pads with different design were prepared (Fig. S2c) and both sides are connected as shown in Fig. S11(a), so that pads on a single side (Front side) could be used for the probe contact, as shown in Fig. S11(c). Here, the size of the pad is designed differently on each side: (1 mm × 1 mm) on the front side, and (3 mm × 1 mm) on the back side. Note that this method (probe connection method 2) will result in two times higher resistance, since the length is twice longer, as shown in Fig. S11(c). For easy comparison with other sintering methods, the measured resistances were divided by two, so that the resistance of 1.2 mm line length could be shown in Fig. 8 of the main text.


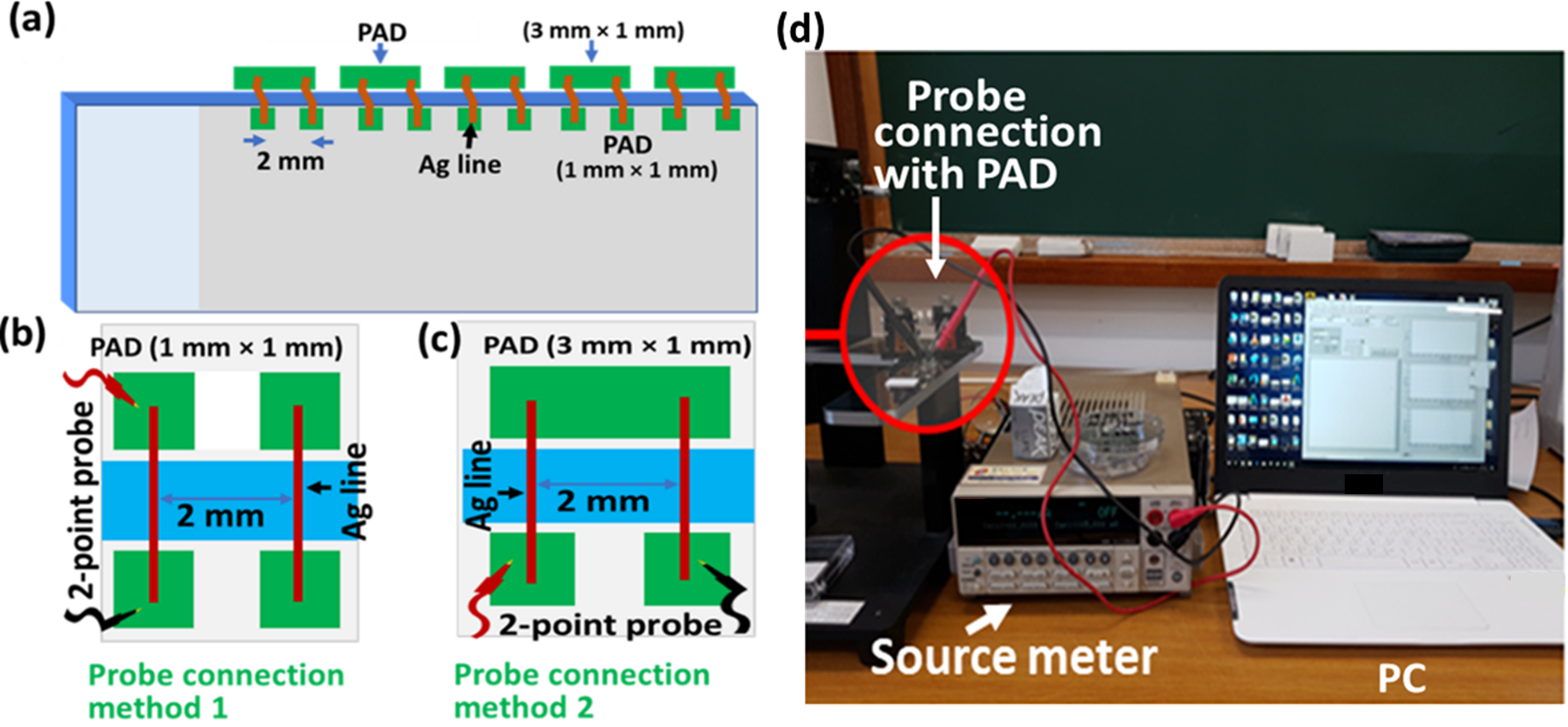


**Figure S11. Experimental set-up for electrical sintering.** (**a)** Schematic of electrode pads for electrical sintering by probing pads on single (front) side only. (**b)** Probing method for pads on both sides (method 1). (**c)** Probing method for pads on single side only (method 2). **(d)** Photo of experimental setup for electrical sintering.

One of the advantages of using electrical sintering is that the method has the capability of process monitoring by measuring the current and voltage in real-time. In addition, printing defects can be detected during the sintering process. For example, lines at the location of localized defects (smaller cross-sectional area) can be disconnected during sintering, due to the intensive current density at the defective part, as shown in Fig. S12(a). The localized damaged part appears brighter in the microscopic images, compared to non-damaged parts, as shown in Fig. S12(b). Note that if other sintering methods are used, the defective printed lines might not be detected.


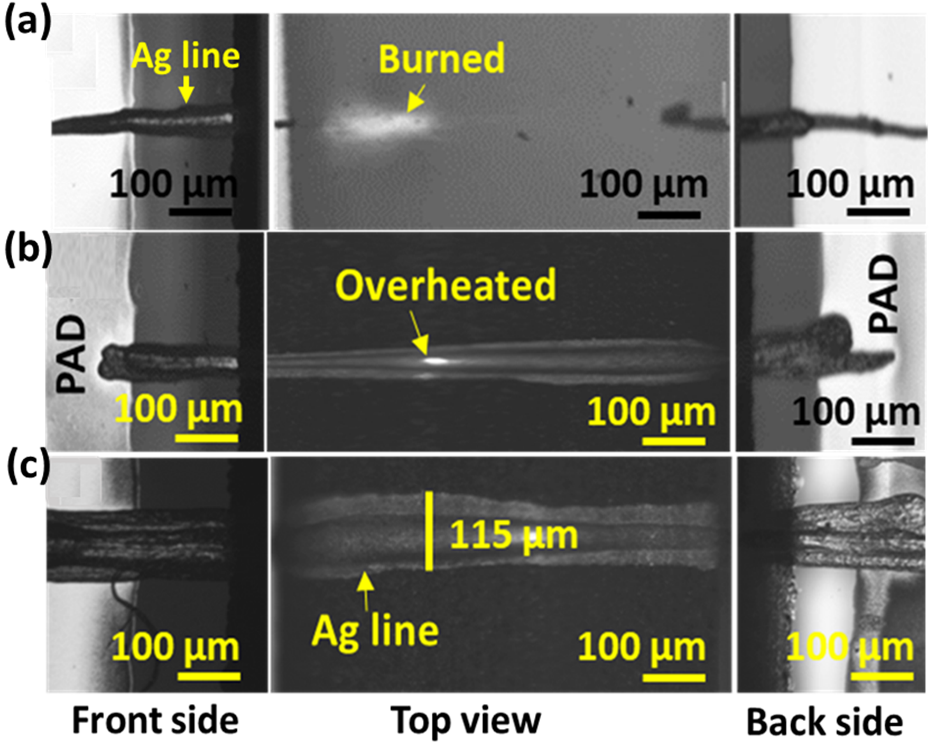


**Figure S12. Optical microscopy images for comparison of damaged and undamaged sintered lines**. (**a)** Disconnected lines due to over current. (**b)** Locally over-heated line. (**c)** Properly sintered line.

*S4.4 Characterization of sintered lines*

After sintering, the resistance between two pads was measured by 4-wire measurement, in order to evaluate the sintered printed lines, as shown in Fig. S13. For this purpose, we used the current source of 100 μA to drive the printed lines. While driving current, the voltage between pads was measured by voltmeter to calculate the resistance, as shown in Fig. S13.


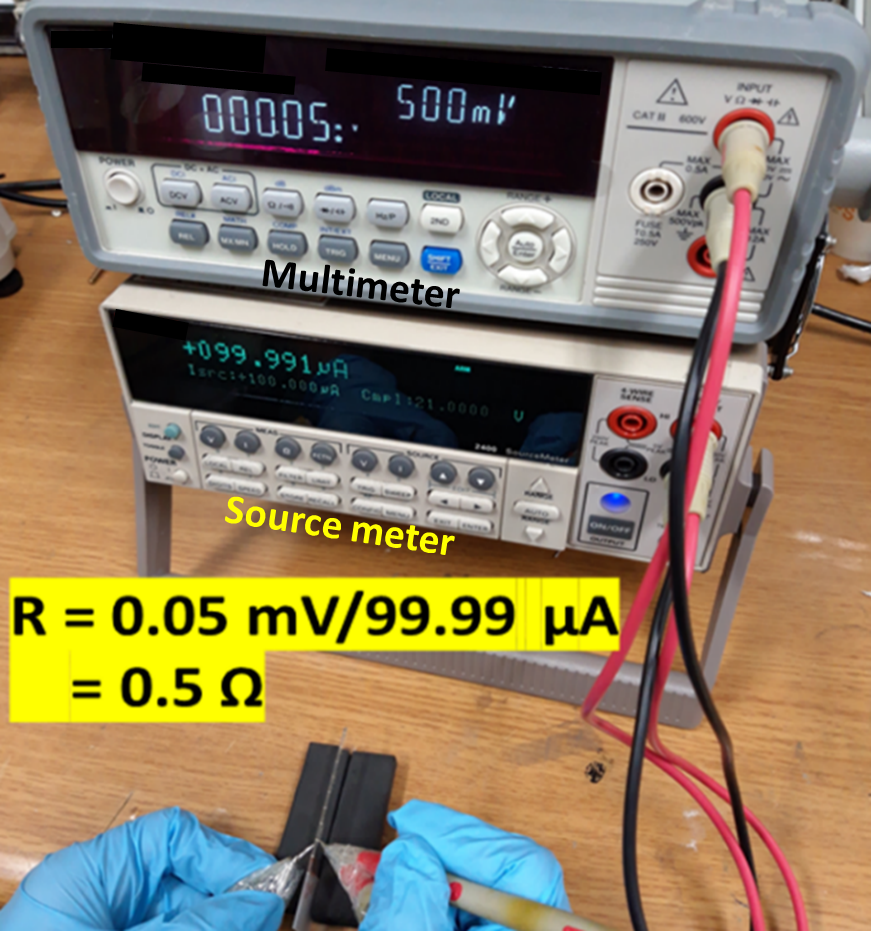


**Figure S13. 4W resistance measurement.**

*S4.5 Comparison of sintering results*

In summary, we compared three different methods of thermal, NIR laser, and electrical sintering via FIB image analysis. Figures S14 (c, e, and g) show that all methods resulted in good connection of the particle grains, even though a few voids were present inside the printed line. Note that the two different layers of printed line and electrode pads are hardly distinguishable, as shown in Figs. S14 (d, f, and h), which indicates that the contact resistance between the pad and printed line can be negligible. The average resistances of printed lines sintered by thermal, NIR laser, and electrical sintering are (0.84, 1.08, and 1.21) Ω, respectively.


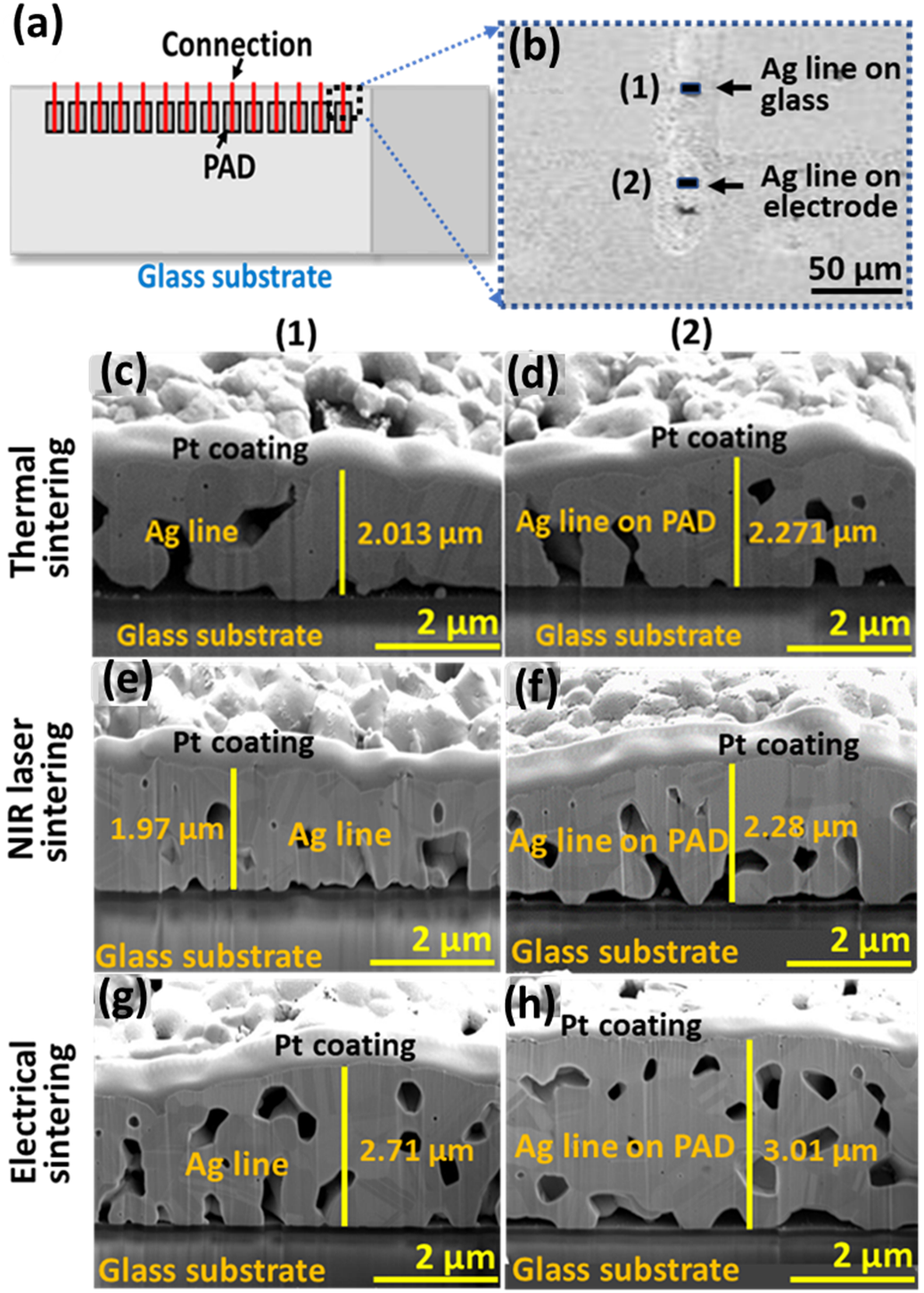


**Figure S14. Cross-sectional FIB image of printed lines**. **(a)** and **(b)** Measurement locations. **(c)** and **(d)** Cross-sectional FIB images of thermal sintered line at locations (1) and (2). **(e)** and **(f)** Cross-sectional FIB images of NIR laser sintered line at locations (1) and (2). **(g)** and **(h)** Cross-sectional FIB images of electrical sintered line at locations (1) and (2).

**Movie:**

**Movie S1:** Printing process of three-dimensional surfaces.
